# Transcriptomic analysis of Chinese yam (*Dioscorea polystachya* Turcz.) variants indicates brassinosteroid involvement in tuber development

**DOI:** 10.3389/fnut.2023.1112793

**Published:** 2023-05-05

**Authors:** Jenny Riekötter, Jana Oklestkova, Jost Muth, Richard M. Twyman, Janina Epping

**Affiliations:** ^1^Department of Biology, Institute of Plant Biology and Biotechnology, University of Münster, Münster, Germany; ^2^Laboratory of Growth Regulators, The Czech Academy of Science, Institute of Experimental Botany and Palacký University, Faculty of Science, Olomouc, Czechia; ^3^Fraunhofer Institute for Molecular Biology and Applied Ecology (IME), Aachen, Germany; ^4^TRM Ltd., Scarborough, United Kingdom

**Keywords:** Chinese yam, *Dioscorea polystachya*, tuber development, brassinosteroids, plant hormones

## Abstract

*Dioscorea* is an important but underutilized genus of flowering plants that grows predominantly in tropical and subtropical regions. Several species, known as yam, develop large underground tubers and aerial bulbils that are used as food. The Chinese yam (*D. polystachya* Turcz.) is one of the few *Dioscorea* species that grows well in temperate regions and has been proposed as a climate-resilient crop to enhance food security in Europe. However, the fragile, club-like tubers are unsuitable for mechanical harvesting, which is facilitated by shorter and thicker storage organs. Brassinosteroids (BRs) play a key role in plant cell division, cell elongation and proliferation, as well as in the gravitropic response. We collected RNA-Seq data from the head, middle and tip of two tuber shape variants: F60 (long, thin) and F2000 (short, thick). Comparative transcriptome analysis of F60 vs. F2000 revealed 30,229 differentially expressed genes (DEGs), 1,393 of which were differentially expressed in the growing tip. Several DEGs are involved in steroid/BR biosynthesis or signaling, or may be regulated by BRs. The quantification of endogenous BRs revealed higher levels of castasterone (CS), 28-norCS, 28-homoCS and brassinolide in F2000 compared to F60 tubers. The highest BR levels were detected in the growing tip, and CS was the most abundant (439.6 ± 196.41 pmol/g in F2000 and 365.6 ± 112.78 pmol/g in F60). Exogenous 24-epi-brassinolide (epi-BL) treatment (20 nM) in an aeroponic system significantly increased the width-to-length ratio (0.045 ± 0.002) compared to the mock-treated plants (0.03 ± 0.002) after 7 weeks, indicating that exogenous epi-BL produces shorter and thicker tubers. In this study we demonstrate the role of BRs in *D. polystachya* tuber shape, providing insight into the role of plant hormones in yam storage organ development. We found that BRs can influence tuber shape in Chinese yam by regulating the expression of genes involved cell expansion. Our data can help to improve the efficiency of Chinese yam cultivation, which could provide an alternative food source and thus contribute to future food security in Europe.

## 1. Introduction

The genus *Dioscorea* contains more than 600 monocotyledonous plant species, most of which grow in the tropics or subtropics of Africa, Southeast Asia, Central America and South America, while a few are native to temperate regions such as North America and Europe (1–3). Some *Dioscorea* species, known as yams, are cultivated for tuber production and are economically important staple food crops in many African countries (4, 5). Chinese yam (*Dioscorea polystachya* Turcz., synonyms: *D. batatas*, *D. pseudobatatas, D. rosthornii*, *D. swinhoei* and *D. opposita*) is native to China, Korea, Taiwan and Kuril Island, and is the only edible yam species that can be grown in temperate regions (4, 6–8). The underground storage organs are derived from the hypocotyl and are rich in starch, protein, fiber and minerals (9–11). They also contain bioactive compounds such as diosgenin and dioscin, which reduce lipid levels in the blood and inhibit the uptake of cholesterol, hence their use in traditional medicine (4, 12, 13). Dioscorin, the major storage protein in Chinese yam, is an antioxidant (14–18). Given these benefits, Chinese yam is also described as functional food (6).

Chinese yam is dioecious but sexual reproduction is rare due to its infrequent and asynchronous flowering (19). Vegetative (clonal) propagation is preferred for cultivation, using seed tubers or aerial tubers (bulbils) that are formed in the leaf axils, for planting (4). Chinese yam has been grown in East Asia for thousands of years, but is nearly unknown in western countries (20). It is also largely overlooked by scientists due to its long life cycle and polyploid genome, which limits the available genetic information (21, 22). Chinese yam has not been adopted in Europe due to the labor-intensive cultivation of the twining vines, which require staking and can grow more than 3 m high (7, 23–25). Furthermore, the club-like or spindle-shaped underground tubers grow up to 1 m deep in the soil due to the positive gravitropism of the actively growing tuber tip, which contains amyloplasts that act as gravity-sensing statoliths to guide tuber formation (26). While growing into the ground, the tuber tip becomes thicker while the head region near the surface remains thin, resulting in a distinctive shape. The head region, as the most mature part of the tuber (23), probably enters dormancy first, characterized by its lacking meristematic activity (6). Dormancy, defined by the absence of visible growth in plant structure containing a meristem (27), is assumed to start right at tuber initiation in yam (28, 29). The tubers are fragile and this shape means they cannot be mechanically harvested or manually pulled out of the soil without breakage (6, 23). Tubers are typically harvested by manual digging, which is an economic challenge for the establishment of this crop in Europe (6).

Brassinosteroids (BRs) are polyhydroxylated steroid hormones that regulate plant cell elongation, cell division, cell differentiation, stress responses and photomorphogenesis (30–32). There are more than 50 naturally occurring BRs that can be classified as C_27_, C_28_ or C_29_ types based on side chain substitutions (33–35). The synthesis of BRs begins when isopentenyl pyrophosphate and dimethylallylpyrophosphate produced by the mevalonate and/or methylerythritol phosphate pathways are converted to cycloartenol, which is then converted to cholesterol, campesterol or sitosterol in the endoplasmic reticulum (35, 36). The 5α-reductase DET2 converts cholesterol to cholestenol, followed by a C22-α-hydroxylation reaction catalyzed by the cytochrome P450 monooxygenase CYP90B1 (DWF4) to produce C_27_-BRs. The same enzymes catalyze analogous reaction steps in the conversion of campesterol to C_28_-BRs or and sitosterol to C_29_-type BRs (33, 37–39). Finally, the BRs are transported to the apoplast where they bind to the membrane-localized receptor BR-INSENSITIVE1 (BRI1), triggering a signaling cascade leading to the dephosphorylation of the transcription factors BRASSINAZOLE-RESISTANT1 (BZR1) and BRI1-EMS-SUPPRESSOR1 (BES1) (31, 40). Dephosphorylated BZR1 and BES1 enter the nucleus and regulate the transcription of genes involved in plant growth and development (41–43).

Exogenous BR application promotes stem growth but inhibits root growth in *Arabidopsis thaliana* (44). However, BRs act in a dose-dependent manner, promoting growth at low concentrations and inhibiting growth at high concentrations (45–47). Brassinolide (BL), the presumed final product of C_28_-BR biosynthesis, is the most biologically active BR followed by its immediate precursor castasterone (CS) (46, 48–50). Given its high bioactivity, BL is usually present at very low concentrations (36, 51). To ensure BR homeostasis, BR biosynthesis is tightly regulated by a negative feedback loop, which is necessary for normal plant development (48, 52).

The disruption of BR synthesis or signaling in *A. thaliana* causes severe dwarfism, a de-etiolated phenotype in the dark, and a dark-green color, as shown for the knockout mutants *det2-1* and *bri1-5* (37, 53). Similar phenotypes were observed in potato (*Solanum tuberosum*) plants following the silencing of *BRI1*, along with a significant reduction in tuber yield and weight per tuber, indicating a role for BR signaling in tuberization (54). The regulation of tuberization and especially tuber shape in *D. polystachya* has not been investigated in detail.

**Figure 1:**
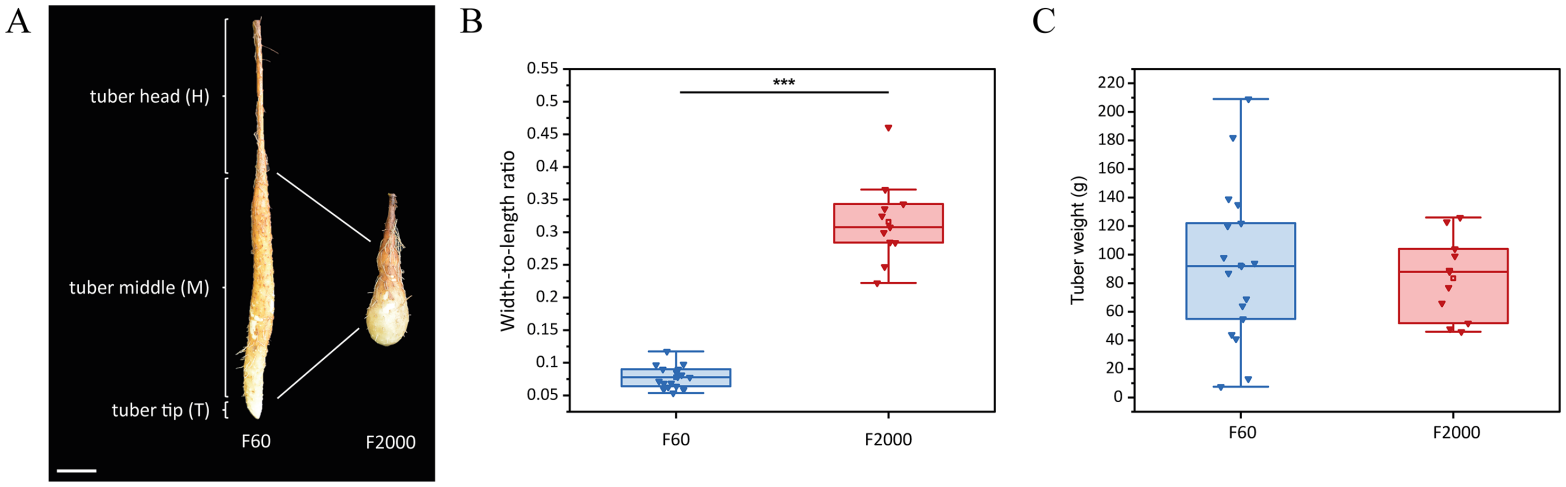
Tuber shape variant F60 (long, thin) vs. F2000 (short, thick) of the Chinese yam (*Dioscorea polystachya*) 3 months after sprouting. **(A)** For transcriptomic comparison, tubers of F60 (left) and F2000 (right) were divided into three parts: the head (H), middle (M) and growing tip (T). Scale bar = 5 cm. **(B)** Length-to-weight ratio and **(C)** weight (g) of harvested tubers (*n* = 17 F60, *n* = 11 F2000). Horizontal lines show medians, box limits indicate the 25^th^ and 75^th^ percentiles, the filled square represents the mean, and lower and upper whiskers represent values differing at least −1.5× the interquartile range (IQR) from the 25th percentile or + 1.5 × IQR from the 75^th^ percentile. Statistical significance was established using a Mann–Whitney U-test (***p < 0.001).

We therefore compared two Chinese yam tuber shape variants: F60, with long but thin tubers, and F2000, with short but thick tubers (Figure 1). F2000 was derived from F60 by vegetative propagation and selection for the preferred tuber shape over a period of several decades. Therefore, F60 and F2000 have a similar genetic background and this should enable the isolation of genetic factors involved in tuber shape development by comparative transcriptomics. Here we present new insights into the network of phytohormones and genes involved in the process of tuber expansion and enlargement in *D. polystachya* by comparing the transcriptomic data of variants F60 and F2000. We also quantified BR levels in *D. polystachya* tubers to determine their impact on tuber shape.

## 2. Materials and methods

### 2.1. Plant materials

Tubers of Chinese yam variants F60 and F2000 were provided by a local farmer (St. Calude de Diray, Loir-et-Cher, France). Plants were cultivated in raised-bed gardens in Münster, Germany (51°57′55.3″N 7°36′54.1″E) from May to December 2018. The tubers were then harvested and placed in cold storage in the dark for least 4 months. For RNA-Seq experiments, pre-season tubers were cut into ~3.5 × 4.5 cm pieces (seed tuber). Raised-bed gardens (1.2 × 0.8 × 1.4 m) in Münster, Germany (51°58′34.8″N 7°35′03.5″E) were filled with a 1:1 mixture of topsoil and silica sand (Dobermann, Münster, Germany). In April 2019, tuber pieces were planted in rows 15 cm apart and were covered with a thin layer of topsoil/sand. After sprouting and vine development, plants were staked to enable normal growth. Plants (*n* = 11 F2000 and *n* = 17 F60) were harvested 3 months after sprouting during the enlargement stage (55). Tubers were washed with tap water, divided into the head (H), middle (M) and tip (T), and flash frozen in liquid nitrogen. Head region was defined by its thin circumference and darker (brown/dark brown) peel. The actively growing tip was recognized by its bright yellow/white peel and cut was approx. made 2 cm from the distal end. Residual tuber part (thicker region and yellow/brown peel) was defined as the tuber middle. Samples were stored at −80°C and freeze dried for at least 4 days. For further analysis, tuber samples were ground to fine powder using an A11 basic analytical mill (IKA-Werke, Staufen, Germany).

Chinese yam cv. Yam 21 plants were provided by the Genebank department of the Leibniz Institute of Plant Genetics and Crop Plant Research, Gatersleben, Germany and were grown in the field in Münster, Germany (51°58′34.8″N 7°35′03.5″E) from May to December 2020 for bulbil production. In the next season, bulbils were grown in the field in Münster to produce mini seed tubers, which were harvested in December 2021. Seed tubers were stored at 4°C in the dark for at least 2 months. For the aeroponic growth experiment, these seed tubers were planted in a 1:1 mixture of topsoil and silica sand in 12 cm × 12 cm × 10 cm pots and cultivated in a phytochamber under long-day conditions (16 h artificial light, 100 μmol m^−2^ s^−1^/8 h darkness) at 25°C/18°C.

### 2.2. *De novo* transcriptome assembly and gene functional annotation

RNA was extracted from yam tuber material for *de novo* transcriptome assembly (Supplementary Methods). Following library construction and Illumina sequencing, at least 50 million 150-bp paired-end reads per sample were obtained. Raw reads were processed to remove adapter sequences, low-quality reads (>50% of bases with a Qphred value <20), and reads of poly N-sequences (*N* > 10%). During this step, we calculated the Q20 and Q30 values and GC content of the clean reads. For *de novo* transcriptome reconstruction, clean reads were assembled using Trinity r20140413p1 (min_kmer_cov = 2, min_glue = 2, all other parameters set by default) (56) and redundant Trinity results were removed using CORSET (57). Genes were annotated against the non-redundant protein sequences (NR), SWISS-PROT and euKaryotic Orthologous Groups and Cluster of Orthologous Groups of proteins (KOG/COG) databases using DIAMOND v0.8.22 (58), nucleotide sequences (NT) database using BLAST v2.2.28+ (59), Protein family (Pfam) database using HMMER 3.1b1 (60), Kyoto Encyclopedia of Genes and Genomes (KEGG) database using KAAS r140224 (61)and Gene Ontology (GO) database using Blast2go b2g4pipe_v2.5 (62). KEGG annotated genes were assigned to KEGG pathways.

### 2.3. Differential expression and enrichment analysis

Differentially expressed genes (DEGs) between pairs of samples were identified using RSEM v1.3.0 to estimate the transcript abundance in each sample (63). Clean reads were mapped to the assembled transcriptome and the read count per gene was calculated based on the mapping results. Additionally, the read counts for each gene in each sample were converted to FPKM values (expected number of fragments per kilobase of transcript sequence per million mapped reads) to estimate gene expression levels by considering different gene lengths and sequencing depths. Differential gene expression was analyzed using the normalization method in DESeq2 v1.10.1 (64). The estimated *p* values were based on the negative binomial distribution, and were adjusted by applying the Benjamini-Hochberg multiple hypothesis testing procedure to control the false discovery rate (65). Genes with an adjusted *p* value (*p*_adj_) < 0.05 were considered as differentially expressed. GOseq v1.32.0 and topGO v2.32.0 were used for GO enrichment analysis (66, 67). KEGG pathway enrichment was analyzed using KOBAS v3.0 to detect the interactions of multiple genes in KEGG pathways (68, 69). Significantly enriched GO and KEGG terms were defined by a corrected *p* value <0.05.

### 2.4. Gene expression analysis by quantitative real-time PCR

DEGs selected for RNA-Seq data validation and BR-regulated gene expression were analyzed by quantitative real-time PCR (qRT-PCR). We included randomly selected as well as BR pathway-specific DEGs for validation. Total RNA was isolated and transcribed into cDNA using the PrimeScript RT Master Mix (Takara Bio Europe, Saint-Germain-en-Laye, France) and qRT-PCR was carried out on a CFX96 Touch Real-Time PCR Detection System (Bio-Rad Laboratories, Hercules, CA, USA) with the KAPA SYBR FAST qPCR Master Mix (2×) Kit (Roche, Basel, Switzerland). The housekeeping genes *DpTUB* and *DpTIP41* were selected for normalization. The qRT-PCR primers designed for each target are listed in Supplementary Table 1. For each target gene, we tested three biological replicates in three technical replicates, each comprising a 10-μL reaction containing 2.5 μL cDNA (diluted 1:20), 5 μL KAPA SYBR FAST qPCR Master Mix (2×) and 2.5 μL mRNA-specific primer mix (2 μM). Each reaction was heated to 95°C for 3 min, followed by 44 cycles of 95°C for 3 s, 60°C for 20 s and 95°C for 5 s. Amplification was confirmed by melt curve analysis in 0.5°C increments from 58 to 95°C. Normalized gene expression levels were calculated using the 2^−ΔΔCt^ method (70).

### 2.5. Extraction and quantification of endogenous BRs

Tuber samples (~5 mg dry weight) were extracted in ice-cold 60% acetonitrile for 12 h at 4°C and 25 pmol of deuterium-labeled internal BR standards was added to each sample (OlChemIm, Olomouc, Czech Republic). After centrifugation (36,670× *g*, 15 min, 4°C), supernatants were loaded onto 50-mg Discovery DPA-6S cartridges (Supelco, Bellefonte, PA, USA), evaporated to dryness, and redissolved in 40 μL methanol for ultrahigh-performance liquid chromatography/tandem mass spectrometry (UHPLC–MS/MS) analysis on an ACQUITY UPLC I-Class system (Waters, Milford, MA, USA) coupled to a Xevo triple quadrupole mass spectrometer (Waters MS Technologies, Manchester, UK) as previously described (34, 71). Each sample was analyzed five times.

### 2.6. Exogenous BR treatment in aeroponic systems

For the exogenous BR treatment experiment, we used *D. polystachya* cv. DpYam 21. This variety produces a greater number of bulbils than F60 and F2000, enabling the sufficient production of mini seed tubers of similar starting material weight. After the germination of Yam 21 mini seed tubers, plants were transferred to an aeroponic system consisting of a box (78 cm × 49 cm × 38 cm) with nozzles connected to a Gardena (Ulm, Germany) garden pump 3500/4 (800 W, 3500 L/h, 4 bar) and a lid containing six holes. Shoots were placed in the holes and held in Rockwool cubes, enabling the mini seed tubers and the new developing tubers to hang inside the aeroponic chamber. We used 12 plants per treatment and the first exogenous treatment was applied after 1 week of acclimation. The newly developed tubers and roots were sprayed with 1/10 strength Murashige and Skoog (MS) medium (Duchefa, Haarlem, Netherlands) including vitamins (pH 5.8) for 10 s every 3 min. For the exogenous BR treatment, we diluted 10 mM 24-epi-brassinolide (epi-BL) in DMSO (APExBIO Technology, Houston, TX, USA) with 1/10 MS to final concentrations of 20 nM or 1 nM, the latter with an adjusted DMSO volume. For mock-treated plants, an equal volume of DMSO was diluted with 1/10 MS. Plants were cultivated in a greenhouse at the Fraunhofer IME, Aachen, Germany under long-day conditions and treated once weekly for 24 h. On other days, plants were sprayed with 1/10 MS medium only. The 8-week-old tubers in the enlargement stage were harvested after the day of the seventh treatment, documented, and immediately frozen in liquid nitrogen for qRT-PCR analysis.

### 2.7. Phenotypic characterization

For the transcriptomic comparison experiment, we measured the tuber weight, length and width. For the epi-BL treatment experiment, we measured the weight, length and width of the newly developed tubers and total root weight. We used ImageJ software^1^ for tuber length and width measurements.

## 3. Results

### 3.1. Phenotypic and genotypic analysis

F60 (long and thin) and F2000 (short and thick) tubers were harvested 3 months after sprouting to confirm the heritable phenotype (Figure 1A). We calculated the width-to-length ratio of the tubers (Figure 1B). A significantly higher ratio was observed for the F2000 tubers (mean = 0.316) compared to the F60 tubers (mean = 0.078) indicating that F60 tubers were longer and thinner. Additionally, no significant difference in tuber weight was observed between the varieties indicating no change in biomass (mean_F2000_ = 83.5 g, mean_F60_ = 92.4 g; Figure 1C). ISAP marker PCR was used to verify the close genetic background of both tuber shape variants (72). We observed an identical pattern for F60 and F2000, but it was distinguishable from other *D. polystachya* cultivars, confirming the close genetic relationship between the tuber shape variants (Supplementary Figure 1).

### 3.2. Transcriptome assembly analysis

To determine the genetic factors responsible for yam tuber shape, we compared the transcriptomes of the F60 and F2000 tubers using three biological replicates per variant. Total RNA was isolated from the dormant head, middle and actively growing tip of each tuber for the preparation and sequencing of cDNA libraries. After quality control, we obtained an average of 59,069,041 (17.7 Gb), 62,292,940 (18.7 Gb) and 68,932,774 (20.7 Gb) cleaned Illumina reads for the head, middle and tip of the F60 tubers, and 65,394,734 (19.06 Gb), 66,156,022 (19.8 Gb) and 57,673,077 (17.3 Gb) cleaned reads for the F2000 tubers (Supplementary Table 2). The Q20 and Q30 scores exceeded 98 and 94%, respectively, and the GC% content was >45%. A *de novo* transcriptome was assembled using Trinity based on the filtered clean reads. This generated 191,270 unigenes with an average length of 1,148 bp (minimum = 201 bp, maximum = 55,357 bp). The N50 length of the assembly was 1807 bp. The largest proportion of unigenes had a sequence length of 200–500 bp (67,551, 35.3%), followed by 500–1,000 bp (50,038, 26.2%) and 1–2 kbp (43,670, 22.8%) (Table 1).

**Table 1:** Statistics and length distribution of the Trinity *de novo* transcriptome assembly.

Length range	Unigene
200–500 bp	67,551 (35.3%)
500–1,000 bp	50,038 (26.2%)
1,000–2,000 bp	43,670 (22.8%)
>2,000 bp	30,011 (15.7%)
Total number	191,270
Min. length	201 bp
Max. length	55,357 bp
Mean length	1,148 bp
N50 length	1,807 bp

### 3.3. Functional annotation and classification

The unigenes were used as queries to screen the NR/NT, SWISS-PROT, Pfam, KOG, KO and GO databases, resulting in the annotation of 67.3% of the unigenes in at least one database (Table 2). A BLASTx search against the NR database led to the annotation of 105,710 transcripts (55.3%, e-value ≤1 × 10^−5^), among which 46.3% demonstrated >80% sequence similarity (Supplementary Figure 2A). The results of the distribution of BLASTx matches by species were in agreement with previous analyses of the yam transcriptome (Supplementary Figure 2B) (73, 74). Based on GO annotations, 47,384 (24.8%) of the unigenes could be assigned to biological process, cellular component and/or molecular function categories (Supplementary Figure 2C). Regarding KO annotations and KEGG pathway classifications, most of the unigenes were sorted to the pathways of translation, followed by carbohydrate metabolism and signal transduction (Supplementary Figure 2D).

**Table 2:** Statistics of unigene functional annotations.

Database	Number of unigenes	Percentage (%)
NR	105,710	55.27
NT	80,140	41.9
KO	51,478	26.91
SWISS-PROT	99,364	51.95
Pfam	75,033	39.23
GO	47,384	24.77
KOG	44,848	23.45
Annotated in all databases	13,068	6.83
Annotated in at least one database	128,765	67.32

NR, non-redundant protein sequences; NT, nucleotide sequences; KO, KEGG Orthology; Pfam, Protein family; GO, Gene Ontology; KOG, euKaryotic Orthologous Groups of proteins.

### 3.4. Analysis of differentially expressed genes by tuber part comparison

**Figure 2:**
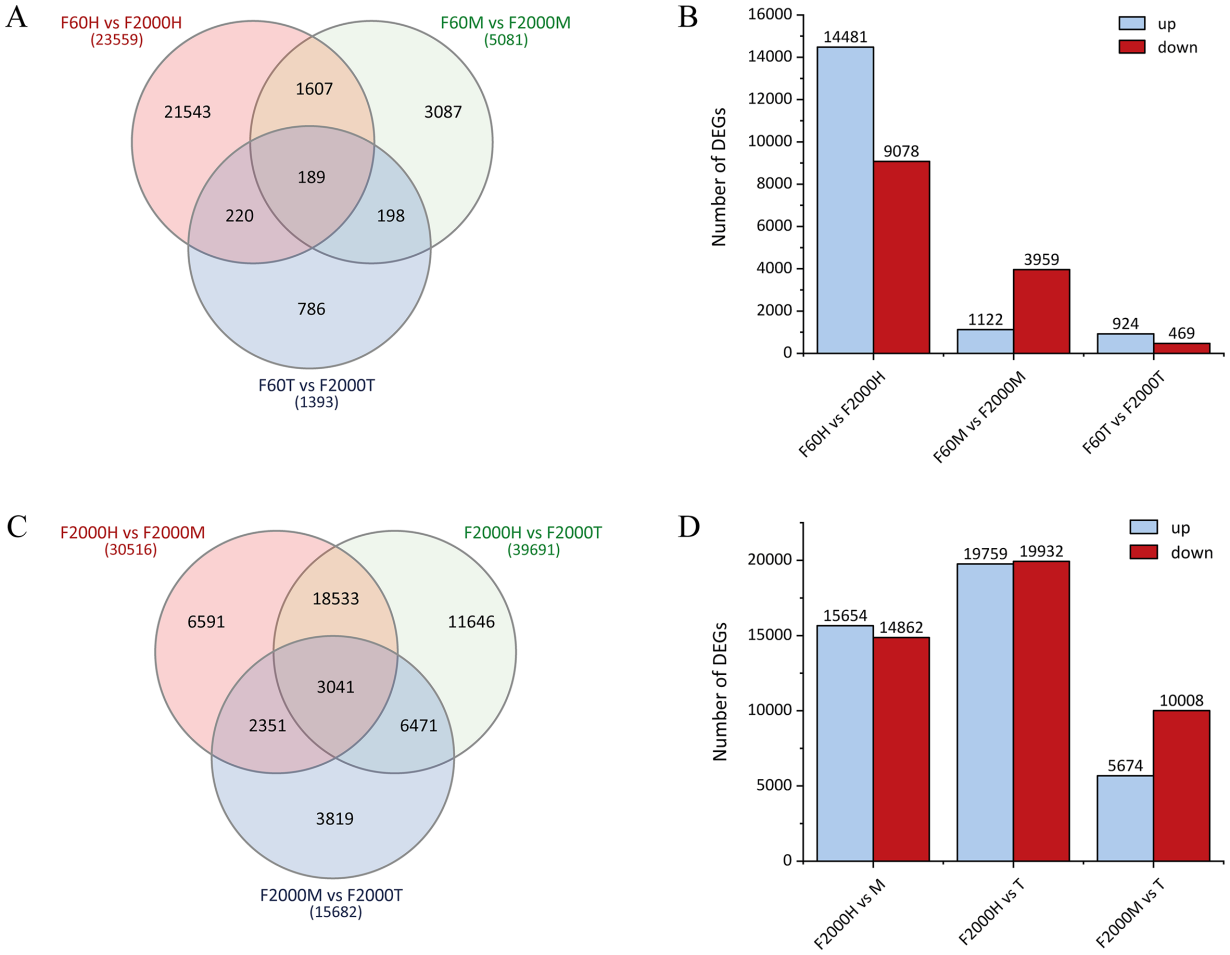
Differentially expressed genes (DEGs) identified by pairwise transcriptome comparisons. **(A)** Venn diagram of DEGs in the tuber head (H), middle (M) and tip (T) when comparing the F60 and F2000 varieties (*p*_adj_ < 0.05) and **(C)** between F2000 tuber parts. **(B)** Numbers of DEGs with higher expression in F60 (up) or F2000 (down) in the tuber head, middle and tip. **(D)** Number of DEGs showing a higher expression in the F2000 tuber head, middle and tip.

Reads were mapped against the *de novo* transcriptome at a rate of >72% for each sample. Pairwise comparisons of expression levels between the F60 and F2000 libraries (Supplementary Table 3) and between the head, middle and tip libraries of F2000 (Supplementary Table 4) revealed a gradient in the number of DEGs from head to tip. The transcriptomic comparison of F60 vs. F2000 led to the identification of 23,559, 5,081 and 1,393 DEGs in the head, middle and tip, respectively (Figure 2A), among which 14,481, 1,122 and 924 were expressed more strongly in the head, middle and tip of the F60 tuber, respectively (Figure 2B). Only 189 genes were differentially expressed in all three parts, 61 of which were more strongly expressed in F2000 and 113 in F60. The greatest interface of DEGs was detected when comparing the heads/middles of the two varieties (F60H vs. F2000H and F60M vs. F2000M), revealing >1,600 DEGs, 1,233 upregulated and 245 downregulated in both parts of F2000. The smallest interface of DEGs was observed when comparing the tuber middle and tip (198 exclusive DEGs). In the pairwise comparisons between F2000 tuber sections, the greatest number of DEGs was identified between the head and tip (Figure 2C). Nearly equal numbers of genes were upregulated and downregulated in these tuber segments, indicating the transcriptomes are distinct, whereas there were few DEGs when comparing the middle and the tip (Figure 2D).

### 3.5. Validation of RNA-Seq data

**Figure 3:**
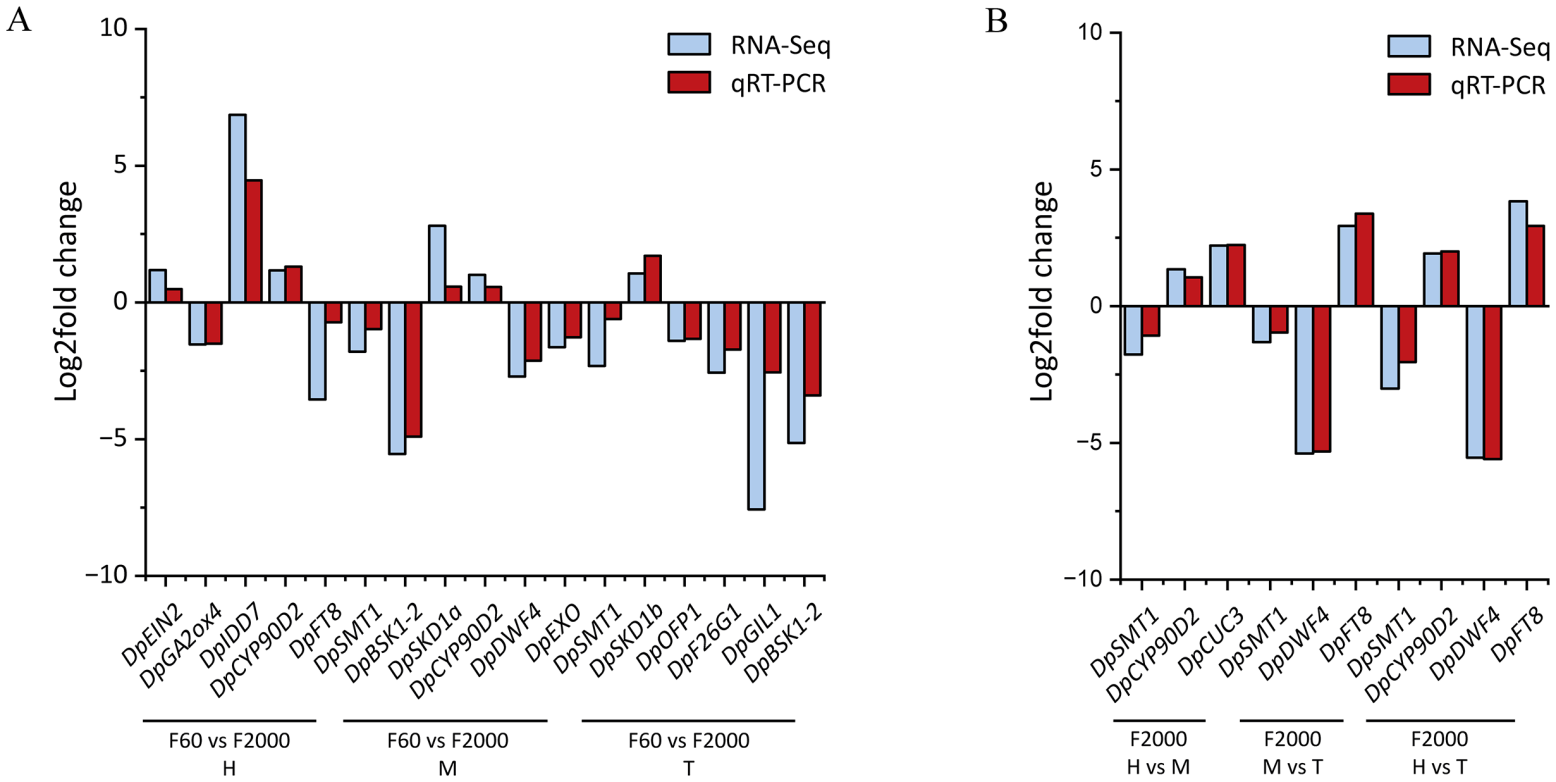
Comparison of relative gene expression (log_2_ fold change) of selected unigenes determined by RNA-Seq and qRT-PCR between **(A)** tuber shape variants F60 and F2000 and **(B)** tuber pats of F2000 (*n* = 3).

The RNA-Seq data were validated by qRT-PCR analysis to confirm the expression profiles of 15 selected DEGs. The qRT-PCR expression profiles when comparing F60 and F2000 samples as well as the tuber parts of F2000 were similar to those determined by RNA-Seq, demonstrating the reliability and accuracy of the RNA-Seq dataset (Figure 3).

### 3.6. GO terms and KEGG pathways related to tuber development

**Figure 4:**
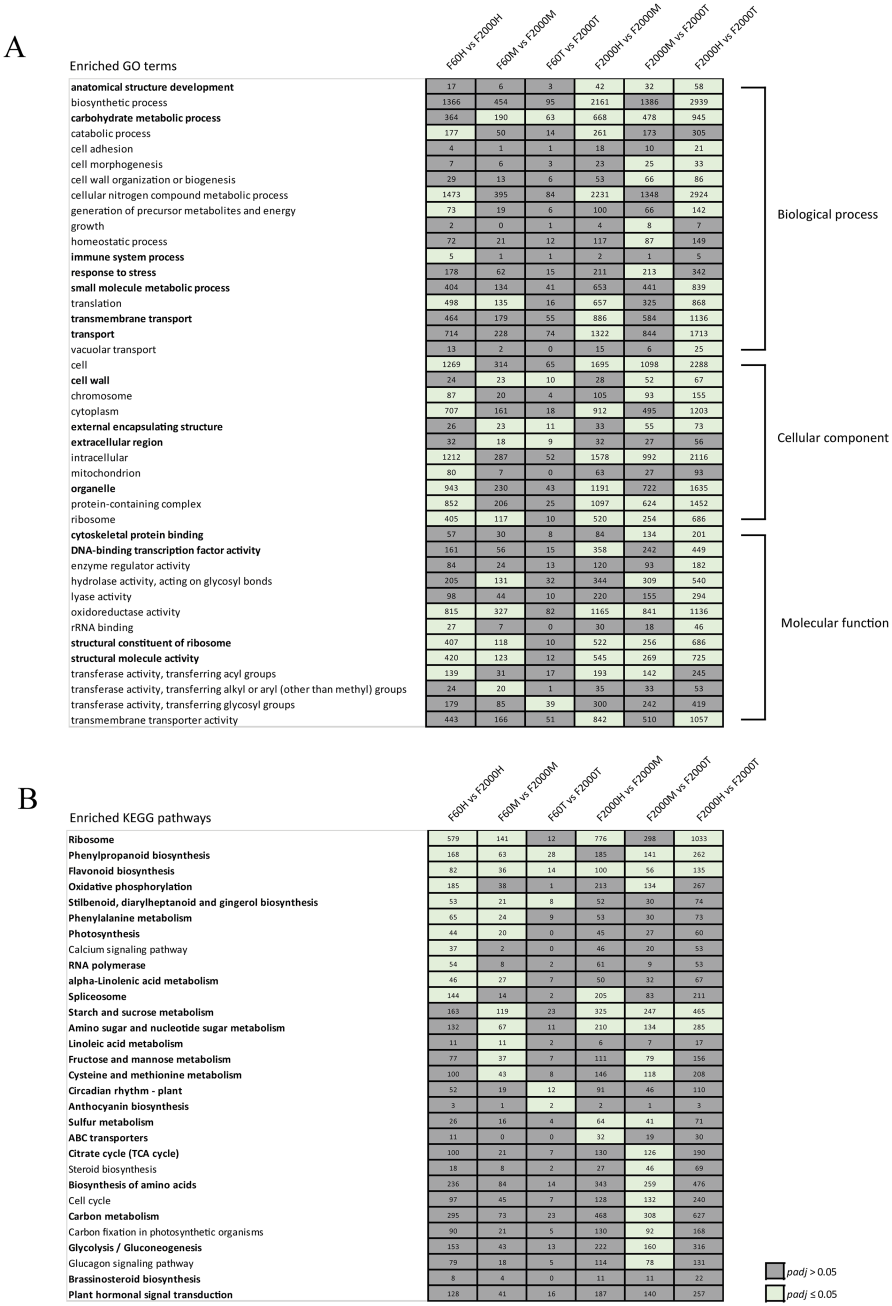
GO term and KEGG pathway enrichment analysis. Selection of **(A)** GO terms and **(B)** KEGG pathways enriched in DEGs. Numbers of DEGs are given in the corresponding cells, and terms related to tuberization are highlighted in bold text. Significantly enriched GO terms and KEGG pathways are indicated by green cells (*p*_adj_ ≤ 0.05).

Although only a small proportion of the unigenes was annotated based on the GO and KO databases, enrichment analysis provided an overview of DEGs assigned to certain GO terms or KEGG pathways and thus refined a list of candidate genes involved in tuber development. The middle and tip comparisons of F60 and F2000 tubers shared the significantly enriched GO terms carbohydrate metabolic process (GO:0005975), cell wall (GO:0005618), external encapsulating structure (GO:0030312), and extracellular region (GO:0005576) (Figure 4A; Supplementary Table 5). These GO terms have also been identified in transcriptomic studies investigating the formation of tubers or tuberous/storage roots in other plant species (75–77) suggesting our DEG candidates related to these GO terms may be involved in Chinese yam tuber development.

In the F2000 tubers, GO terms related to carbohydrate metabolic process, oxidoreductase activity (GO:0016491) and anatomical structure development (GO:0048856) were significantly enriched in all three comparisons (Supplementary Table 6). The GO terms cell wall organization or biogenesis (GO:0071554) and cell morphogenesis (GO:0000902) were significantly enriched in the F2000H vs. F2000T and F2000M vs. F2000T comparisons.

KEGG pathway enrichment analysis revealed three secondary metabolic pathways that were enriched in all three comparisons between F60 and F2000 tubers: “phenylpropanoid biosynthesis” (ko00940), “flavonoid biosynthesis” (ko00941) and “stilbenoid, diarylheptanoid and gingerol biosynthesis” (ko00945) (Figure 4B; Supplementary Table 7). Most DEGs in the phenylpropanoid and flavonoid pathways showed higher expression levels in F2000H and F2000M but lower levels in F2000T. In the F2000 tubers, DEGs representing these two pathways were significantly enriched in the middle vs. tip and head vs. tip comparisons. Whereas most DEGs were upregulated in the F2000H vs. F2000T comparison, DEGs were more strongly expressed in the F2000T vs. F2000M samples (Supplementary Table 8). The phenylpropanoid biosynthesis pathway, which is also involved in lignin biosynthesis (78), and flavonoid biosynthesis pathway have been linked to storage organ formation in tuberous crops such as sweet potato (*Ipomoea batatas*), cassava (*Manihot esculenta*) and *Cynanchum auriculatum* (76, 79–82). Genes involved in lignin biosynthesis could therefore be particularly important for Chinese yam tuber enlargement because declining expression levels are thought to control the transition from fibrous roots to storage roots in sweet potato (76).

#### 3.6.1. Sugar and starch metabolism

The KEGG term “starch and sucrose metabolism” (ko00500) was significantly enriched in the F60M vs. F2000M comparison. The genes strongly expressed in the F2000M samples included several related to sucrose and starch metabolism (e.g., *DpSUS1* and *DpSUS2*), *fructokinase* (*DpFRK*) genes, *glucose-1-phosphate adenylyltransferase* (*DpAPS1*) genes and *starch synthase* (*DpSS1-4*) genes. However, seven unigenes related to *DpSUS4* were exclusively expressed in F60M. In contrast, no starch synthase genes were detected in the tuber tip comparison, and *DpSUS1* and *DpSUS4* were expressed at higher levels in F60T. In the F2000 transcriptome, starch and sucrose metabolism was significantly enriched in all three comparisons between the tuber sections. Here, genes related to starch biosynthesis and cellulose degradation were upregulated in a gradient toward the tuber tip. Several endoglucanase and β-xylosidase genes were upregulated in the F2000T samples, where they presumably facilitate the hydrolysis of cellulose and xylan, respectively, during tuber morphogenesis (83, 84).

#### 3.6.2. Circadian clock

Several DEGs were linked to the “circadian rhythm – plant” pathway (ko04712). Although most of these DEGs were detected in the F60H vs. F2000H comparison, the KEGG pathway was significantly enriched only in the F60T vs. F2000T comparison. Here, the majority of DEGs were expressed at higher levels in F60. We identified two-component response regulator-like genes (*DpPRR37*) that were expressed more strongly in F60 than F2000 in all three tuber parts, whereas *phytochrome A* (*DpPHYA*) genes were only expressed more strongly in the head of F60 tubers. We also identified genes encoding CONSTANS (DpCO) and FLOWERING LOCUS T-like (DpFT) proteins, which regulate flowering as well as tuberization (85, 86). In the tuber head, three of the seven *DpCO* genes and five of the seven *DpFT-like* genes showed higher expression levels in F2000. In contrast, all differentially expressed *DpCO* genes in the middle and tip comparisons were expressed at higher levels in F2000, whereas three *DpFT-like* genes were more strongly expressed in the F60T samples. Genes encoding Adagio protein 3 (DpADO3/DpFKF1) were expressed at higher levels in F2000M and F2000T. Importantly, *DpFT* and *DpCO* gene expression were upregulated in a gradient toward the head in F2000 tubers, whereas several *DpPRR37* genes were mainly expressed in F2000M. In addition, genes encoding the transcription factors LATE ELONGATED HYPOCOTYL (DpLHY) and PHYTOCHROME-INTERACTING FACTOR 3 (DpPIF3) were upregulated in F2000H, whereas *phytochrome B* (*DpPHYB*) genes were upregulated in F2000T. Phytochromes are photoreceptor proteins that sense light and regulate plant growth accordingly (87). We also identified a *GRAVITROPIC IN THE LIGHT 1* (*DpGIL1*) gene, which was expressed more strongly in F2000 than F60 in all three tuber parts. This protein plays a role in phytochrome-mediated agravitropism enabling randomized hypocotyl growth in *A. thaliana* (88).

#### 3.6.3. Hormonal pathways involved in tuber shape

##### 3.6.3.1. Jasmonic acid and its derivatives

We found that “α-linolenic acid metabolism” (ko00592), which includes jasmonic acid (JA) biosynthesis, was also significantly enriched in the two upper tuber parts. In the F60H vs. F2000H comparison, we detected 46 DEGs potentially involved in this pathway, 33 of which were expressed at lower levels in F60. A similar tendency was observed in the F60M vs. F2000M comparison, where 25 of 27 DEGs were expressed more strongly in F2000. Although not significantly enriched, two genes encoding 13-lipoxygenase (DpLOX), potentially catalyzing the oxygenation of linoleic acid, were expressed at higher levels in F60T, whereas two genes encoding a hydroperoxide dehydratase (DpAOS) and a 12-oxophytodienoic acid reductase (DpOPR) were expressed more strongly in F2000T. In the F2000 tuber, numerous DEGs involved in JA biosynthesis were upregulated in a gradient toward the tuber head, particularly genes encoding OPR, 4-coumarate-CoA ligase (DpOPCL1) and acyl-coenzyme A oxidase (DpACX), as well as three genes encoding jasmonic acid-amido synthetase (DpJAR1). These data point to a role for JA and its derivatives in the upper part of the tubers.

##### 3.6.3.2. Auxins

We found that genes encoding the auxin-responsive proteins DpIAA30, DpIAA25 and DpIAA17 were expressed at higher levels in the F2000M samples. These transcriptional repressors dimerize with AUXIN RESPONSE FACTOR (DpARF) proteins to inhibit auxin-regulated gene expression (89). The *DpARF9* and *DpARF15* genes were expressed at higher levels in F60M and F2000M, respectively. Other auxin-responsive genes with higher expression in F2000M included *Gretchen Hagen3* (*DpGH3*) and *small auxin upregulated RNA* (*DpSAUR*). Several genes involved in auxin signal transduction were also upregulated in F2000T compared to F2000M, including seven *DpAUX1*, three *DpTIR1*, 25 *DpIAA*, 17 *DpARF*, five *DpGH3* and five *DpSAUR* genes. We also identified a gene encoding a transmembrane kinase 4 (DpTMK4/BARK1), which was more strongly expressed in F2000T than F60T, and in the F2000 tip compared to the head and middle samples. AtTMK4 plays an important role in auxin signaling and the auxin-mediated growth of *A. thaliana* plants (90).

##### 3.6.3.3. Gibberellins

Gibberellins (GA) are key phytohormones required for potato tuberization (91). The F60M vs. F2000M and F60T vs. F2000T pairwise comparisons revealed no DEGs involved in GA signaling. Moreover, no genes involved in GA biosynthesis were differentially expressed in the tip between the variants. In contrast, genes encoding ent-kaurene oxidase (DpGA3) and gibberellin 3-β-dioxygenase 2 (DpGA3ox2) as well as a *gibberellin 20 oxidase 1* (*DpGA20ox1*) gene were expressed at higher levels in the F2000M samples, whereas one *DpGA20ox1* gene was more strongly expressed in the F60M samples. Four *ent-kaurenoic acid oxidase* (*DpKAO*) genes and six *gibberellin 2-β-dioxygenase* (*DpGA2ox*) genes were expressed at higher levels in the F2000H samples, while six *DpGA2ox1* genes were more strongly expressed in the F60H samples. Other components of GA signaling were differentially expressed in the F60H vs. F2000H comparison, including *gibberellin receptor GID1C-like* (*DpGID1*) with higher expression in the F60H samples. The F2000H vs. F2000T comparison revealed the upregulation of *KAO* and *GA20ox1* in the tuber head, whereas *DpGA3* and *DpGA3ox* were upregulated in the tip. Strikingly, a *DpGA2ox* gene was upregulated in the tuber tip, whereas two additional *DpGA2ox* genes were upregulated in the head, potentially resulting in the inactivation of GA (92).

##### 3.6.3.4. Brassinosteroids

In the intersection of DEGs between F60M vs. F2000M and F60T vs. F2000T, we identified several genes related to or potentially regulated by BR signaling that were expressed at higher levels in the F2000 samples. These encoded serine/threonine kinases such as DpBSK1, BSK1-2 and DpCDL1, and the cell wall-related proteins EXORDIUM-like (DpEXO) and DpXTH9. Genes encoding the ovate family protein (DpOFP) were expressed at higher levels in all three F2000 tuber parts. In the middle and tip comparisons, we also identified several *DpEXPA* genes encoding cell-wall related expansin proteins. In contrast, the *CUP-SHAPED COTYLEDON 3* (*DpCUC3*) gene, which defines organ boundaries (93), was more strongly expressed in F60T. The DEGs involved in BR biosynthesis included three *sterol 24-C-methyltransferase 1* (*DpSMT1*) genes, one *methylsterol monooxygenase 1* (*DpSMO1*) gene, one *cycloeucalenol cycloisomerase* (*DpCPI1*) gene, and two *δ(24)-sterol reductase-like* (*DpDWF1*) genes with higher expression levels in F2000M vs. F60M. These enzymes represent the steroid biosynthesis pathway leading to campesterol, the precursor of C_28_-BRs. Additionally, the *DpCYP90B1* (*DpDWF4*) and *DpCYP90A1* (*DpCPD*) genes, encoding enzymes that catalyze downstream reactions, showed higher expression levels in the F2000M samples, whereas a *CYP90D2* gene was expressed more strongly in the F60M samples. Interestingly, a *DpCYP734A1* unigene, potentially involved in BR inactivation and homeostasis (94), was expressed more strongly in the F2000M samples, as was the gene encoding a potential BZR1 homolog. In the F2000M vs. F2000T comparison, the steroid biosynthesis pathway was significantly upregulated in the tuber tip. Subsequently, we found that nine *DpDWF4* genes were also upregulated in the tuber tip, along with genes encoding the BR receptor complex BAK1/BRI1, a *DpBSK* gene, and a gene encoding a TETRATRICOPEPTIDE THIOREDOXIN-LIKE 1 (DpTTL1) protein. In *A. thaliana*, these proteins are thought to be involved BR signaling and may play a role in cell expansion (95, 96). Finally, we found that BR-responsive genes such as *DpEXO*, *DpTCH4* and *DpCYCD3* were downregulated in the tuber middle. These data suggest that BR synthesis is more active in the tuber tip, potentially increasing the BR content.

### 3.7. Elevated BR content in tuber tips

Based on the transcriptomic data described above, we evaluated the BR content and composition in the F60 and F2000 tubers, revealing higher levels of castasterone (CS), 28-norCS, 28-homoCS and brassinolide (BL) in F2000 tuber tissues (Table 3). Peak levels of BRs were detected in the actively growing tuber tip, with CS the most abundant and BL the least abundant in both variants. Significant differences in 28-norCS levels between F60 and F2000 tubers were detected in the tip, whereas the most striking differences in the middle were observed for 28-homoCS and CS levels. A significantly higher level of 28-homoCS was detected in the F60 tuber tip compared to the middle.

**Table 3:** Endogenous BR levels in Chinese yam tubers (pmol g^−1^ DW ± SEM).

	F60			F2000		
	Head	Middle	Tip	Head	Middle	Tip
CS	5.59 ± 1.49 ab	5.6 ± 1.27 a	365.57 ± 112.78 ab	20.26 ± 11.75 ab	22.12 ± 7.65 b	439.62 ± 196.41 ab
28-norCS	3.68 ± 0.71 ab	3.13 ± 1.09 ab	27.49 ± 6.67 a	6.94 ± 1.03 ab	6.64 ± 2.35 ab	45.81 ± 1.73 b
28-homoCS	2.42 ± 0.76 abc	0.93 ± 0.29 a	77.30 ± 20.80 bc	6.14 ± 1.86 abc	6.66 ± 2.39 c	111.25 ± 13.53 abc
BL	1.74 ± 0.84 a	2.35 ± 0.52 a	9.42 ± 1.75 a	4.91 ± 1.73 a	3.05 ± 0.77 a	16.00 ± 2.27 a

CS, Castasterone; BL, Brassinolide. Different letters indicate significant differences in compound content between the tuber shape variants or tuber parts (Dunn’s post hoc test after Kruskal-Wallis, statistics on rows, *p* < 0.05, *n* = 3).

### 3.8. Epi-BL treatment increases the tuber width

**Figure 5:**
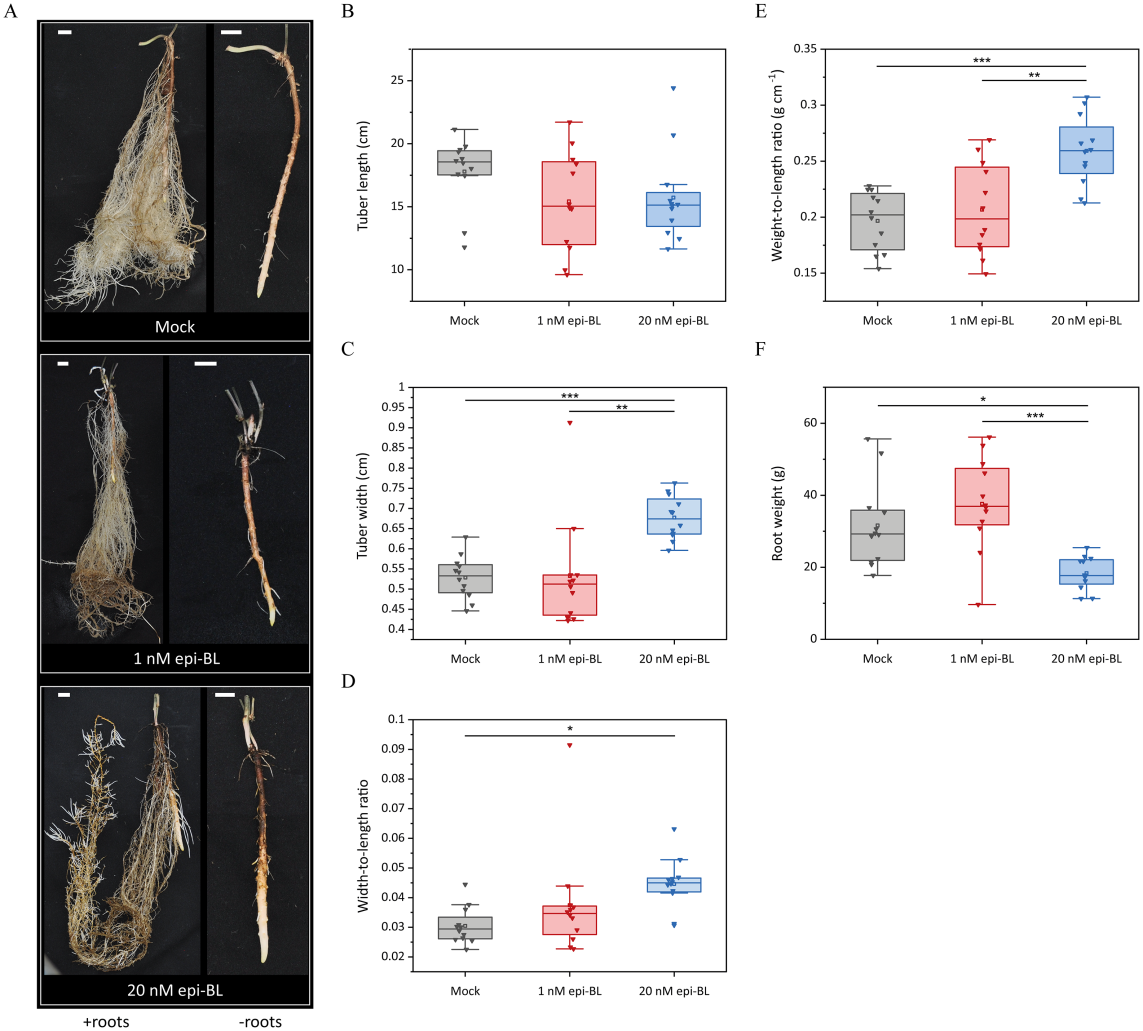
Effect of exogenous epi-brassinolide (epi-BL) on Chinese yam (*Dioscorea polystachya* cv. Yam 21) tubers in aeroponic systems. Tubers were treated weekly with 1 nM epi-BL, 20 nM epi-BL or DMSO (Mock) for 24 h. **(A)** After 7 weeks of treatment, tubers and roots were harvested (left – with roots; right – magnification and removed roots). Scale bar = 1.5 cm. **(B,C)** Tuber width and length were measured and **(D)** width-to-length and **(E)** weight-to-length ratios were calculated. **(F)** Total root weight was measured. Horizontal lines show medians, box limits indicate the 25th and 75th percentiles, the filled square represents the mean, and lower and upper whiskers represent values differing at least −1.5× the interquartile range (IQR) from the 25th percentile or + 1.5 × IQR from the 75th percentile. Asterisks indicate statistically significant differences based on one-way ANOVA followed by Tukey’s *post hoc* test (***p < 0.001, **p < 0.01, *p < 0.05, *n* = 12).

We investigated the effect of BRs on tuber development *in planta* by applying exogenous epi-BL to Chinese yam tubers in an aeroponic system. Tubers were treated with 1 or 20 nM epi-BL once a week for 24 h. After 7 weeks, the tubers in the 20 nM treatment group were shorter (15.725 ± 1.034 cm) than those of the mock-treated plants (17.794 ± 0.792 cm) (Figures 5A,B). A significant increase in tuber width was observed in the 20 nM treatment group (0.667 ± 0.015 cm) compared to the mock (0.529 ± 0.015 cm) and 1 nM (0.533 ± 0.039 cm) treatment groups (Figure 5C). The width-to-length ratio of the mock-treated tubers (0.03 ± 0.002) was therefore significantly lower compared to the 20 nM epi-BL treatment group (0.045 ± 0.002) (Figure 5D). Due to the tuber shape of Chinese yam, we also calculated the weight-to-length ratio as an indicator of shorter but thicker tubers. In the 20 nM treatment group, the weight-to-length ratio was significantly higher (0.259 ± 0.009 g cm^−1^) compared to the 1 nM (0.207 ± 0.012 g cm^−1^) and mock (0.197 ± 0.008 g cm^−1^) treatment groups (Figure 5E). We also observed differences in root morphology and weight between the treatments. In contrast to the bright roots of the control plants, the roots treated with epi-BL were browner and showed a significant weight loss in the 20 nM treatment group (18.358 ± 1.325 g) compared to the 1 nM treatment group (37.63 ± 3.741 g) and mock-treated plants (31.608 ± 3.412 g) (Figures 5A,F).

### 3.9. Effect of epi-BL treatment on the expression of RNA-Seq candidate genes

**Figure 6:**
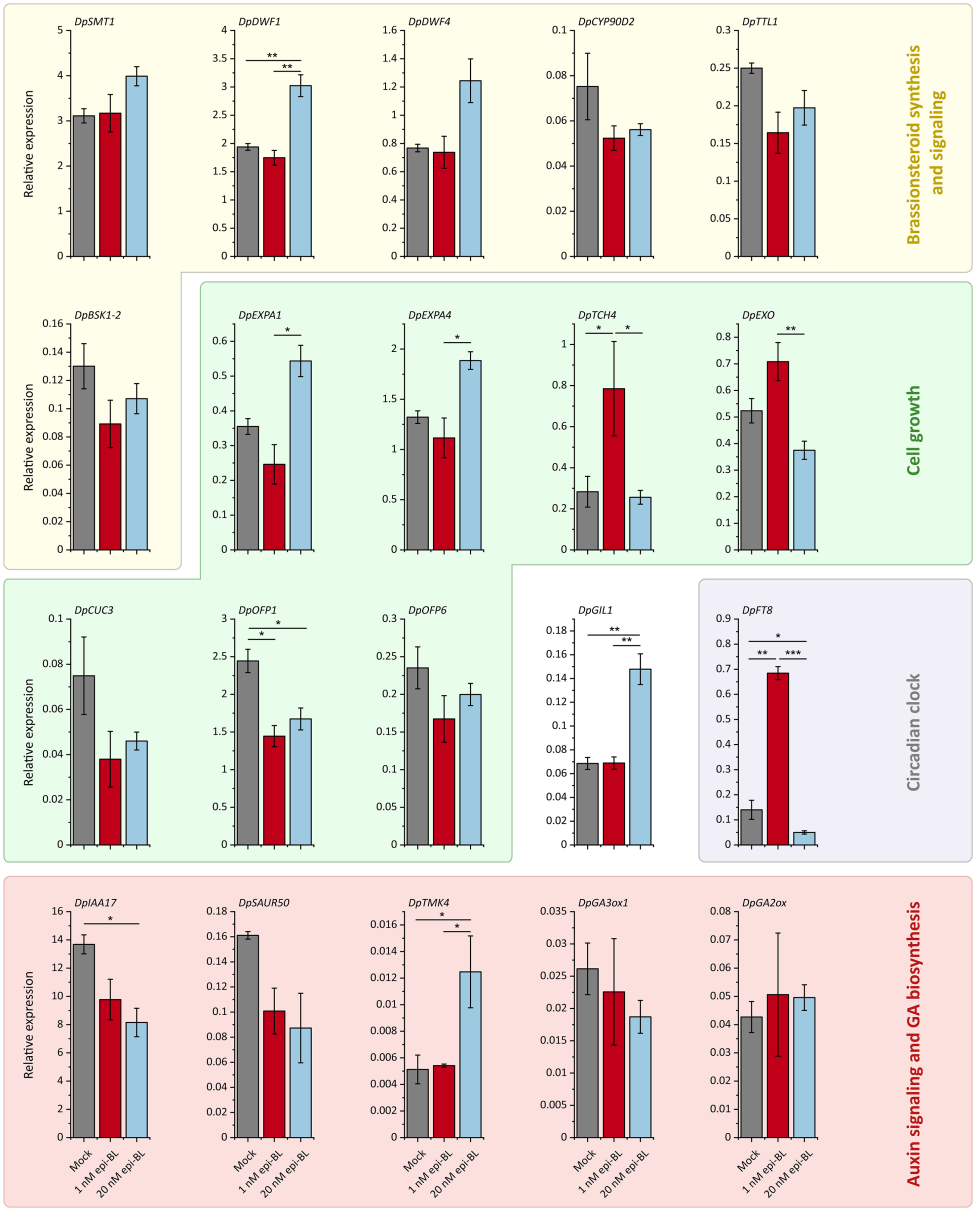
Effects of exogenous epi-brassinolide (epi-BL) treatment (1 nM and 20 nM) on the relative expression of RNA-Seq candidate genes in Chinese yam tubers. After 7 weeks of treatment with 1 nM epi-BL, 20 nM epi-BL or DMSO (Mock), relative gene expression levels were determined by qRT-PCR. Data are means ± SEM of three pools each consisting of four biological replicates. Asterisks indicate statistically significant differences based on one-way ANOVA followed by Tukey’s *post hoc* test (***p < 0.001, **p < 0.01, *p < 0.05, *n* = 12).

Previous studies in other plants have shown that exogenous BR generally downregulates the expression of genes involved in BR synthesis due to negative feedback (36, 97–101). Therefore, we investigated the effect of exogenous BR on gene expression in Chinese yam tubers. In our aeroponic experiment, the relative expression levels of steroid and BR biosynthesis-related genes were affected by the epi-BL treatment. We observed the upregulation of *DpSMT1*, *DpDWF1* and *DpDWF4* in the 20 nM treatment group, while *DpCYP90D2* was downregulated in both treatment groups compared to the control (Figure 6). In contrast, the expression of *DpTTL1* and *BSK1-2*, both encoding proteins with roles in BR signaling, was suppressed by 1 and 20 nM exogenous epi-BL.

We also investigated the expression of genes from our transcriptomic comparisons if previous studies have shown that homologs in other species are regulated by BR signaling (102–104). The expression of cell growth-related genes was affected in a dose-dependent manner. The *DpEXPA1* and *DpEXPA4* genes were induced in the 20 nM treatment group but suppressed in the 1 nM treatment group compared to the control. In contrast, *DpEXO* and *DpTCH4* were induced by 1 nM epi-BL but suppressed or unaffected at the higher concentration. Interestingly, *DpOFP1, DpOFP6* and *DpCUC3* expression was inhibited in both epi-BL treatment groups compared to the mock treatment control. The auxin signaling-related genes *DpIAA17* and *DpSAUR50*, which showed higher expression toward the F2000 tuber tip in RNA-Seq experiments, were suppressed by the epi-BL treatment in a dose-dependent manner, whereas *DpTMK4* expression significantly increased in the 20 nM treatment group compared to the mock-treated plants and the 1 nM epi-BL treatment had no effect. We observed a slight dose-dependent reduction in *DpGA3ox1* expression and a slight increase in *DpGA2ox* expression but these changes were not statistically significant.

We also tested the effect of epi-BL treatment on *DpGIL1*, which was expressed at higher levels in all F2000 tuber parts compared to F60. The 20 nM epi-BL treatment induced *DpGIL1* expression by more than twofold, whereas the 1 nM treatment had no significant effect. Finally, we investigated the expression of the circadian clock-related gene *DpFT8*, which was downregulated in the F2000 tuber tip compared to the head and middle and also showed a higher expression in F2000H vs. F60H (Figure 3). The 1 nM epi-BL treatment significantly upregulated *DpFT8* expression by more than fourfold, whereas the 20 mM treatment caused significant downregulation compared to the control and the 1 nM treatment group.

## 4. Discussion

Several previous studies have focused on the genetic basis of tuber initiation and expansion in *D. polystachya* (55, 73, 105) but the control of tuber shape is poorly understood, despite its major impact on the efficiency of mechanical harvesting (6, 106). By comparing the transcriptomes of two closely related tuber shape variants (F60 and F2000) each divided into three tuber sections, we identified several candidate genes involved in tuber development. Contrary to our expectations, most DEGs identified when comparing F60 and F2000 were expressed in the assumed dormant tuber head (Figures 2A,C). The largest number of DEGs was identified when comparing the F2000H vs. F2000T samples, suggesting high transcriptional activity in the head as well as distinctive transcriptomes in the upper and lower parts of the tuber.

The KEGG pathway “stilbenoid, diarylheptanoid and gingerol biosynthesis” was significantly enriched in all three comparisons between F60 and F2000. In ginger, these pathways are thought to produce bioactive compounds such as volatile oils, gingerol and diarylheptanoids with antioxidant, anti-inflammatory and anticancer activity (107). Phenylpropanoid and flavonoid biosynthesis also contribute to the formation of pharmacologically active metabolites in ginger (107, 108). Accordingly, these pathways may also result in the production of bioactive compounds such as phenanthrenes (109, 110), which are associated with the health-promoting effects of Chinese yam tubers. Additionally, the enzymes of the phenylpropanoid biosynthesis pathway play a crucial role in lignin biosynthesis by supplying lignin monomers (78). Lignin is the major structural component of the cell wall, providing mechanical strength and support, thus playing an important role in growth (78, 111). Transcriptomic analysis of tuberous crops has suggested a link between phenylpropanoid/lignin biosynthesis and storage organ formation (76, 79–81, 112). In sweet potato and cassava, phenylpropanoid and lignin biosynthesis genes are downregulated during storage root development, indicating that lignin depletion is required for the transition of fibrous to storage roots (76, 82). The lower lignin content presumably enables cell expansion and consequently lateral tuber swelling during storage root formation (112). Therefore, the lower expression of phenylpropanoid biosynthesis genes we observed in F2000T vs. F60T samples may also affect the lignin content, enabling lateral tuber enlargement. However, inconsistent results were obtained regarding DEGs involved in this pathway in the F2000 tuber comparisons. Most of the DEGs were upregulated in the head compared to the middle and tip, but most of the downregulated DEGs were detected in the middle compared to the tip suggesting the lignin content is highest in the thin head region followed by the actively growing tip rather than the middle.

Photoperiodic perception plays an important role in flowering as well as tuberization and is linked to the pseudo-response regulators (PRRs) that control the circadian clock via feedback loops (113, 114). PRRs upregulate *FT* expression directly or by stabilizing CONSTANTS (CO), thus enhancing the binding of CO to the *FT* promoter. Moreover, AtPRR7 was shown to repress the expression of *AtCDF1*, which in turn encodes a repressor of *AtCO* (115). This increases the abundance of *FT* transcripts and promotes earlier flowering (116). In contrast, the rice ortholog of *AtPRR7* (*OsPRR37*) represses the expression of *Heading Date 3A* (*Hd3a*), which is an ortholog of *A. thaliana* FT, resulting in the suppression of flowering (117). In potato, *StPRR* is upregulated in the early-tuberization cultivar Z3 but downregulated in cultivar Z18, which has a longer tuberization time, suggesting a role in tuberization time control (118). PRR proteins can therefore act as activators or repressors by regulating *FT* expression (119). Interestingly, *DpPRR37* was expressed at higher levels in all three parts of the F60 tuber. We also detected higher levels of *DpCO* and *DpFKF1* mRNA in the F2000 middle and tip samples vs. corresponding F60 samples. In potato, under long-day conditions, StFKF1 and StGI form a complex with StCDF1, resulting in StCDF1 degradation (120, 121). Because StCDF1 inhibits the expression of *StCO*, the depletion of StCDF1 leads to the accumulation of *StCO* mRNA, which in turn leads to the transcriptional activation of *StSP5G* (encoding a repressor of the tuberigen StSP6A), resulting in delayed tuberization (85, 121, 122). Contrary to the pro-floral transition activity of *AtCO*, *StCO* represses tuberization (121–124). The higher expression of *DpPRR37* in F60 vs. F2000 may be responsible for the lower expression of *DpFT*, suggesting that DpPRR37 represses *DpFT* as reported in rice (118). In the F2000 tubers, we observed that *DpFT* genes were expressed more strongly in the head, while *DpPRR37* expression was higher in the other tuber parts, supporting the hypothesis that DpPRR37 is a suppressor. Additionally, *DpCO* in F2000 tubers may induce *DpFT* expression. FTs can induce or suppress tuberization, so the effect of *DpPRR37*, *DpCO* and *DpFT* expression on tuber initiation in Chinese yam should be investigated in more detail.

KEGG pathway analysis showed the enrichment of DEGs related to JA biosynthesis. Most of these genes showed higher expression in F2000 tuber parts, including *DpAOS*, which encodes a hydroperoxide dehydratase (allene oxide synthase), the key enzyme catalyzing the dehydration of 13-(*S*)-hydroperoxylinolenic acid in this pathway (125, 126). Multiple genes related to JA biosynthesis were also upregulated in the F2000 tuber head compared to the tuber tip. Additionally, *DpJAR1* genes were strongly expressed in a gradient toward the upper tuber. JAR1 catalyzes the conjugation of isoleucine to JA, forming an important and active JA derivative, and positively regulates JA signaling (127, 128). These results suggest there is a higher JA content in the upper tuber. JAs influence storage organ formation, including the promotion of tuberization in potato (129–131) and yam (132–134). Moreover, endogenous JA levels in potato were shown to peak at tuber set (135). The application of exogenous JA to *D. alata* and *D. cayenensis-D. rotundata* cuttings *in vitro* induced earlier microtuber formation (133, 136). The authors speculated that JA promoted yam tuberization during the initiation phase but had no effect later in development. In potato, exogenous JA stimulates tuber formation, but some studies suggest that JAs instead influence subsequent tuber enlargement by controlling the reorientation of cortical microtubules (137, 138). Interestingly, overexpression of *StJAZ1-like*, a negative regulator of the JA response, suppressed tuber initiation and reduced the average number and weight of potato tubers (139). Therefore, JAs might influence tuber induction in Chinese yam. *DpJAZ10* transcripts were more abundant in all parts of the F2000 tuber. In *A. thaliana*, the perception of JA results in the degradation of JAZ protein, enabling the transcription of JA response genes (140). Simultaneously, *AtJAZ* expression is induced by JA as part of a negative feedback loop (140, 141). Furthermore, several *DpJMT* genes were upregulated in the F2000 lower tuber parts compared to the head. In potato, the overexpression of *StJMT* increased the tuber size and yield (142). *JMT* encodes the jasmonic acid carboxyl methyltransferase that catalyzes the formation of methyl jasmonate (143). This derivative may also influence the enlargement of the lower part of Chinese yam tubers, as reported in potato. Taken together, the higher expression levels of JA biosynthesis genes in the F2000 tuber head and middle could affect the timing of tuber emergence, whereas other phytohormones may regulate tuber enlargement at later developmental stages.

BRs may also regulate tuber enlargement and shape. BRs are negative regulators of shoot gravitropism, control the gravitropic response in *A. thaliana* roots (46, 144–146), and influence plant architecture by regulating cell elongation, cell division and cell differentiation (147). BRs also promote lignin biosynthesis by inducing key genes of the phenylpropanoid pathway, thereby accelerating wound healing in potato (148). Exogenous BR treatment expanded the diameter of beetroot (*Beta vulgaris*) roots by increasing the size of parenchyma cells between the cambial rings and increasing the area of secondary xylem (149). Moreover, the analysis of tuberization in sweet potato roots and tuberous kohlrabi stems revealed enriched DEGs associated with BR biosynthesis at the early stages of root swelling and tuberous stem formation, respectively (150, 151). In our pairwise comparisons, we observed the upregulation of several steroid biosynthesis genes in a gradient toward the tuber tip, as well as *DpDWF4* encoding the key enzyme in BR biosynthesis, regulating endogenous bioactive BR levels (36). The expression of BR signaling components was upregulated in the F2000 tuber tip, suggesting the presence of elevated BR levels. The quantification of endogenous BRs revealed peak levels in the tuber tip, confirming this hypothesis. Additionally, the pairwise comparison of F60 and F2000 confirmed the higher BR content of the F2000 tubers. In other plant species, high BR levels typically occur in young and actively growing tissues, coinciding with highest *DWF4* expression levels (36, 152, 153). In Chinese yam tubers, the tuber tip is the actively growing part, so our results agree with the literature.

Interestingly, BL has not been detected in rice and it is assumed that CS is the end product of BR biosynthesis in some monocotyledonous species. This is because rice and other monocots only possess a single copy of the *CYP85* gene, whereas two copies are present in the dicot *A. thaliana*, one of which evolved the required function for BL synthesis (51). Although, CS seems to be the main BR in *D. polystachya* tubers, a small quantity of BL was detected in all F60 and F2000 tuber parts. The higher expression of a *DpCYP734A1* toward the F2000 tuber tip as well as in F2000M compared to F60M might be required to maintain BR homeostasis. The encoded protein is potentially involved in BR inactivation (94). Given that the effects of BRs on plant growth are highly dose-dependent and already visible at very low concentrations, the amount of active BL is usually small (36, 45–47, 51). Therefore, the inactivation of CS and BL by DpCYP734A1 might ensure optimal growth in yam tubers. The exogenous application of CS was shown to increase the gravitropic response in maize roots (46), while BL treatment disrupted the negative gravitropism of soybean shoots (154). The higher CS content could thus affect the gravitropic response in the Chinese yam tuber tips, influencing the growth direction in a manner still to be investigated.

BRs are known to regulate the expression of numerous genes that control the cellular processes underlying plant growth (41–43, 155). Our RNA-Seq experiments revealed the expression of several cell-wall related genes including *DpEXO*, *DpXTH*, *DpTCH4* and *DpEXPA*. In *A. thaliana*, these genes are induced by BR treatment via the activity of the transcription factors AtBZR1 and AtBES1 (41, 102, 155–157). *AtEXO* overexpression lines showed more prolific vegetative growth, whereas knockout mutants exhibited dwarfism with low biomass production due to the inhibition of cell expansion (156, 158). Furthermore, the overexpression of *EgPHI-1*, which belongs to the PHI-1/EXO/EXL family, resulted in higher root volume without affecting root length in *Eucalyptus globulus* (159). Interestingly, several *DpEXO* genes were upregulated during the enlargement stage compared to the initiation stage of Chinese yam tubers (73). In contrast, the expression of *AtCUC3* was repressed by BRs in *A. thaliana* (104). The stronger expression of *DpCUC3* in F60T vs. F2000T may reflect the elevated BR content in F2000, potentially downregulating *DpCUC3* expression. CUC3 controls organ boundaries by the repression of cell division (160). Our results indicate that endogenous BR levels may regulate cell division and enlargement in Chinese yam, and the higher activity toward the tuber tip could be responsible for the distinctive tuber shape. Furthermore, the higher rate of BR biosynthesis during early tuber development could favor a thicker tuber shape and shortening of the head region. To test whether BRs favor a thicker tuber shape, we treated *D. polystachya* tubers with different concentrations of epi-BL. This resulted in higher width-to-length ratio in the 20 nM treatment group compared to the 1 nM treatment group and control, confirming that epi-BL influences tuber shape. Interestingly, the expression of the sterol and BR biosynthesis genes *DpSMT1*, *DpDWF1* and *DpDWF4* was induced by the 20 nM treatment compared to the control, whereas the 1 nM treatment did not affect their expression. In agreement, our transcriptomic data indicate higher mRNA levels for BR biosynthesis genes in the tuber tip albeit the elevated BR content. However, our findings disagree with other studies showing that exogenous BR usually downregulates *DWF4* and other BR biosynthesis genes in a negative feedback loop (36, 48, 52, 161–164). These differences may reflect the dose-dependent nature of the response. Even so, *DpCYP90D2* expression was suppressed by both epi-BL treatments, in agreement with our transcriptomic data. Here, the lower BR content in the F2000 tuber head was accompanied by higher *DpCYP90D2* expression levels compared to the tuber tip. In rice, the expression of *D2* (*OsCYP90D2*) was also downregulated by BL (163). How the modulation of these genes affects the BR content of Chinese yam tubers remains to be determined, but our data confirm the effects of exogenous BR on the expression of genes required for sterol and BR biosynthesis.

We found that BRs also modulate the expression of cell wall-related genes such as *DpEXPA1*, *DpEXPA4*, *DpEXO* and *DpTCH4*. The expression of *DpEXO* was induced by 1 mM epi-BL but suppressed at 20 mM, whereas the expansin genes showed the opposite profile. These effects may be related to the dose-dependent effects of BRs on plant growth (45–47). Consistent with our RNA-Seq data, *DpCUC3* expression was downregulated by exogenous epi-BL. Therefore, endogenous BRs may regulate cell wall-related gene expression in Chinse yam tuber tips. EXO proteins may cooperate with XTH and TCH4, which modify xyloglucans in the cell wall, thus controlling cell wall loosening (157, 165) to facilitate lateral tuber expansion, whereas CUC3 would inhibit this process.

The OFP family is known to regulate organ shape and may fulfil a similar role in Chinese yam. The overexpression of *SlOFP20* in the tomato (*Solanum lycopersicum*) variety Yellow Pear resulted in rounder fruits, whereas knockdown of the same gene in *S. pimpinellifolium* LA1589 (with an *ovate* background that produces round fruits) produced an elongated fruit shape (166). Furthermore, the tuber shape QTL *Ro* in potato (identified by fine mapping of the F1 cross between the elongated tuber parent DM1-3 and the round tuber parent M6) is regulated by StOFP20 (167–169). Finally, the overexpression of *RsOFP2.3* reduced hypocotyl length in radish (*Raphanus sativus*) but increased hypocotyl width in *A. thaliana* (170). Moreover, *OsOFP1* and *OsOFP8* were induced by BL treatment in rice (103, 171). Therefore, the upregulation of *DpOFP1* and *DpOFP6* in F2000 tubers may reflect the higher BR content, thus repressing tuber elongation while increasing the F2000 tuber width, resulting in a higher width-to-length ratio compared to F60 tubers. However, exogenous BL at both tested concentrations caused the downregulation of *DpOFP1* and *DpOFP6*. These results confirm that BR regulates these genes, although contrary to our expectations and previous reports. It is important to state that high BL concentrations (0.1–1 μM BL) were needed to induce *OsOFP1* and *OsOFP6* in rice, whereas lower concentrations did not affect *OsOFP1* expression compared to controls (103, 171). Moreover, time course-dependent induction of *OsOFP6* was observed after BL treatment, suggesting that *DpOFP* may also be regulated in a dose-dependent manner, and a higher concentration of epi-BL may be required to induce *DpOFPs* in Chinese yam.

The *DpGIL1* gene was expressed more strongly in F2000 vs. F60 (all tuber parts). The function and mode of action of this protein is poorly understood. In *A. thaliana*, AtGIL1 is required for the phytochrome-mediated randomized growth of hypocotyls under low light conditions as demonstrated by seedlings of the *gil1-1* mutant that grew upwards after red or far-red light exposure, thus being unable to overcome the response to gravity (88). BRs regulate the gravitropic response during shoot and root growth, so we also tested the effect of epi-BL treatment on *DpGIL1* expression. We found that 20 nM epi-BL strongly induced *DpGIL1*. Although the Chinese yam tuber tip grows toward gravity, the upregulation of *DpGIL1* may affect the gravitropic response causing the shorter tuber phenotype. The role of BRs and DpGIL1 in the gravitropism of Chinese yam tubers should be studied in future experiments.

Crosstalk between BRs, other phytohormones and the circadian clock has been shown to affect the growth of dicots and monocots (172, 173). Our RNA-Seq data revealed the stronger expression of *DpOPR* in F2000 vs. F60 tubers. The expression of *AtOPR3* was induced by JA and BR, linking these two phytohormone signaling pathways in *A. thaliana* (174). Moreover, BRs are assumed to act upstream of GA by regulating genes encoding key biosynthetic enzymes (175). The transcription factors BES1 and BZR1 bind to the promoter of the *AtGA20ox1* gene, and exogenous BRs strongly increased the expression of *AtGA20ox1* in the *cpd* mutant, partially rescuing its phenotype. Exogenous BR also induced the expression of *D18/OsGA3ox-2* in rice, increasing the GA level and thus regulating cell elongation (176). In contrast, excessive exogenous BR inactivated GA by inducing *OsGA2ox-3* expression. In potato, GAs are thought to regulate tuberization by acting as a mobile signal (114). Whereas high GA levels in the stolon tip promote stolon elongation, low GA levels favor tuberization (91). Interestingly, the application of high concentrations of GA to Chinese yam inhibited bulbil formation but promoted new tuber growth, whereas low concentrations induced bulbil formation and enlargement (177, 178). Although a single application of GA at a low concentration resulted in the greatest tuber yield, higher GA concentrations were required during cumulative treatments to achieve the same effect (134) indicating that minor changes in the hormone concentration have a major impact on plant development. In *D. polystachya* cv. Guihuai 16, endogenous GA_3_ and GA_4_ levels peaked 90 days after planting, coinciding with the rapid growth phase of tuber enlargement, but decreased rapidly after this time point (55). Moreover, the transcriptomic comparison of tuber initiation and expansion in Guihuai 16 plants indicated the presence of an auxin, GA and ABA signaling network. Several *AUX/IAA*, *SAUR*, *ARF* and *GID2* genes were downregulated during the expansion stage, whereas *DELLA* and *PP2C* were upregulated (73). In contrast, we did not detect any differentially expressed *DELLA* genes when we compared F60 and F2000 tubers. However, *GID1* genes were upregulated in the F2000H vs. F60H, F2000H vs. F2000M and F2000H vs. F2000T comparisons, and two *DELLA* genes were upregulated in the F2000M vs. F2000H comparison. Because our tubers were harvested 3 months after sprouting, GA levels may have been declining already. Although we identified several DEGs potentially encoding enzymes involved in GA biosynthesis, including Ga20ox, Ga3ox and the inactivation enzyme GA2ox, no DEGs were detected in the F60T vs. F2000T comparison, indicating a minor role in vertical tuber enlargement. Furthermore, we did not observe a consistent expression pattern among the different F2000 tuber parts because genes annotated as GA20ox and GA2ox were upregulated in the F2000M vs. F2000T comparison. This agrees with a previous report suggesting the outcome was the result of feedback mechanisms or other hormones influencing tuber growth (55, 105). The varied response of GA2ox family enzymes to GA_3_ treatment may reflect their different physiological roles, or may reflect different organ and tissue types (105). In our study, we detected a slight decrease in *DpGA3ox1* expression and an increase in *DpGA2ox* expression following epi-BL treatment, but these changes were not significant compared to the control group. In rice, low BL concentrations (0.1–10 nM BL) significantly suppressed *OsGA2ox-3* expression, whereas higher concentrations (1–10 μM BL) induced this gene (176). In contrast, *OsGA3ox-2* was induced in a dose-dependent manner in the same study. This concentration-dependent effect on GA biosynthesis genes cannot be ruled out in Chinese yam. GAs may therefore be involved in tuber enlargement but are probably not the master regulators of tuber shape in Chinese yam, but rather a component of the complex crosstalk between phytohormone pathways.

Our transcriptomic comparisons revealed several DEGs related to auxin signaling that were expressed at higher levels in F2000 tips vs. other parts, including *DpIAA*, *DpSAUR* and *DpTMK4*. In *A. thaliana*, these and other auxin signaling genes are regulated by auxins and BRs (144, 179–184). IAAs act as negative regulators of auxin signaling by interacting with the transcription factor ARF, whereas SAUR proteins and TMK4 are both involved in the acidification of the cell wall, enabling cell growth (90, 185–187). We found that the exogenous application of 20 nM epi-BL significantly suppressed the transcription of *DpIAA17* while simultaneously inducing *DpTMK4*, indicating crosstalk between BR and auxin signaling in Chinese yam that may orchestrate cell expansion and finally tuber growth.

In *A. thaliana*, exogenous BR promoted the transcription of *FLOWERING LOCUS C* (*AtFLC*), a repressor of floral transition, thus delaying flowering and reducing the flowering rate (188, 189). However, the interaction between AtCO and AtBIN2 indicates that BR also has a positive role in the floral transition (190). AtBIN2 was shown to repress flowering by inhibiting the formation of AtCO dimers and phosphorylating the AtCO protein, which abolishes its DNA-binding activity and thus its ability to induce *AtFT* expression. These findings highlight the ambiguous function of BRs during flowering, and it is likely that BR signaling is integrated with other environmental cues to regulate the floral transition (173). We found that *DpFT8* expression was significantly induced by 1 nM epi-BL but significantly repressed by 20 nM epi-BL, indicating a regulation of *FT* expression by BRs in Chinese yam tubers.

**Figure 7:**
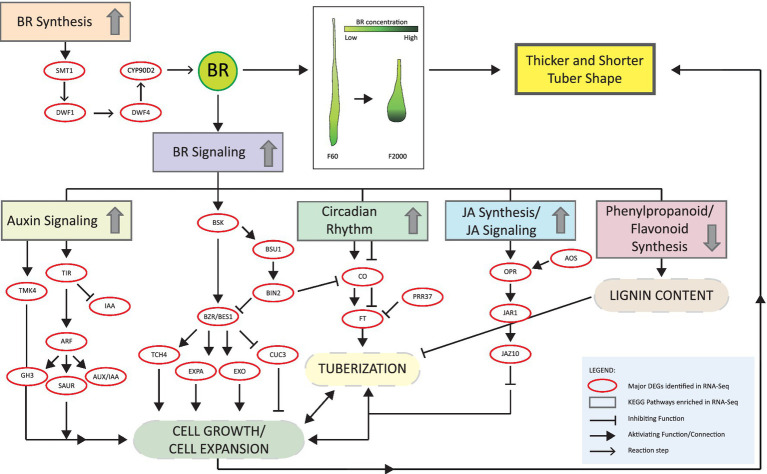
Proposed role of BR network in *D. polystachya* (Chinese yam) tuber growth.

Taken together, our data indicate that the shape of Chinese yam tubers depends on a complex network of phytohormone signaling in which BRs play the central role (Figure 7). To our knowledge, this is the first report describing endogenous BR levels in Chinese yam tubers. We identified CS as the major endogenous BR, and found that its abundance increased toward the tip, supporting the hypothesis that BRs contribute to the specific tuber shape. The increased width and weight-to-length ratio of tubers exposed to epi-BL indicates a shorter tuber that retains the normal amount of biomass, highlighting the role of BRs in tuber growth and morphology. The tuber shape is an important property for yam cultivation because it determines whether economical mechanical harvesting can replace labor-intensive manual harvesting. We found that treatment with epi-BL produced shorter and thicker tubers that are more compatible with mechanical harvesting. Increasing the endogenous BR content of Chinese yam may therefore produce rounder tubers (shorter and thicker, shorter head region). Our findings thus offer a clearer insight into tuber development in *D. polystachya* and provide the basis for crop improvement in future breeding programs.

## Data availability statement

The original contributions presented in the study are publicly available. This data can be found at: https://www.ncbi.nlm.nih.gov/bioproject/PRJNA942579.

## Author contributions

JR, JO, JM, and JE conceived and designed the experiments. JR, JO, and JM performed the experiments. JR, JO, and JE analyzed the data. JR, RMT, and JE wrote the paper. All authors read and approved the manuscript.

## Funding

The junior research group of Chinese yam cultivation in Europe was funded by the German Federal Ministry of Education and Research (grant number 031B0202). JO was supported by an ERDF grant for the project “Plants as a tool for sustainable global development” (no. CZ.02.1.01/0.0/0.0/16_019/0000827).

## Conflict of interest

RMT was employed by TRM Ltd.

The remaining authors declare that the research was conducted in the absence of any commercial or financial relationships that could be construed as a potential conflict of interest.

## Publisher’s note

All claims expressed in this article are solely those of the authors and do not necessarily represent those of their affiliated organizations, or those of the publisher, the editors and the reviewers. Any product that may be evaluated in this article, or claim that may be made by its manufacturer, is not guaranteed or endorsed by the publisher.

## Footnotes

^1^ http://rsb.info.nih.gov/ij/
