# Supplementary material for: Transcriptomic analysis of Chinese yam (Dioscorea polystachya Turcz.) variants indicates brassinosteroid involvement in tuber development
Source: Front Nutr. 2023 May 5;10:1112793. doi: 10.3389/fnut.2023.1112793 (PMC10196131; doi:10.3389/fnut.2023.1112793)
Supplement: Supplementary file 2 [file Data_Sheet_2.pdf]

Supplementary Material

Transcriptomic analysis of Chinese yam (*Dioscorea polystachya* Turcz.) variants indicates brassinosteroid involvement in tuber development

Jenny Riekötter, Jana Oklestkova, Jost Muth, Richard M. Twyman and Janina Epping *

*** Correspondence:** Janina Epping: Janina.epping@uni-muenster.de

1.1 Supplementary Figures

A B

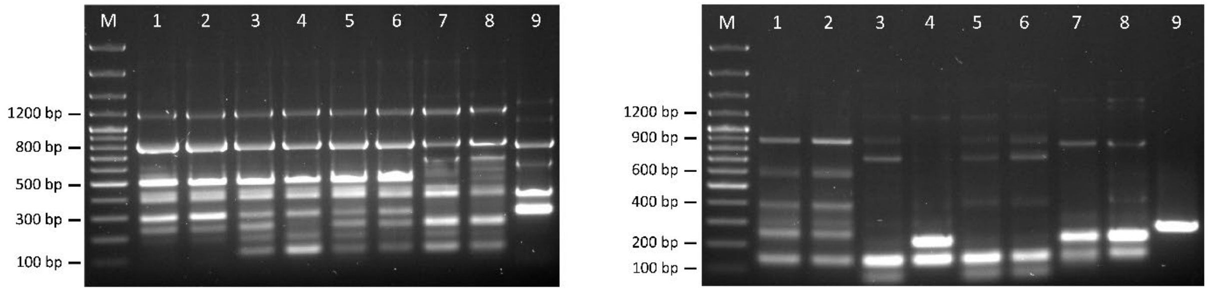

Supplementary Figure 1. ISAP marker analysis for the genotypic characterization of *D. polystachya* cultivars using primer pairs (A) F1R1 and (B) F7R1. M = 100 bp plus marker (Thermo Fisher Scientific), lanes 1–8 = *D. polystachya* ‘F2000’, ‘F60, ‘Nagaimo’, ‘DpYam21’, ‘Genkotsujiro’, ‘Zenguritaro’, ‘Shintanmaru’ and ‘Tanbayamaimo’, lane 9 = *D. rotundata* cv. TDr95/19177.

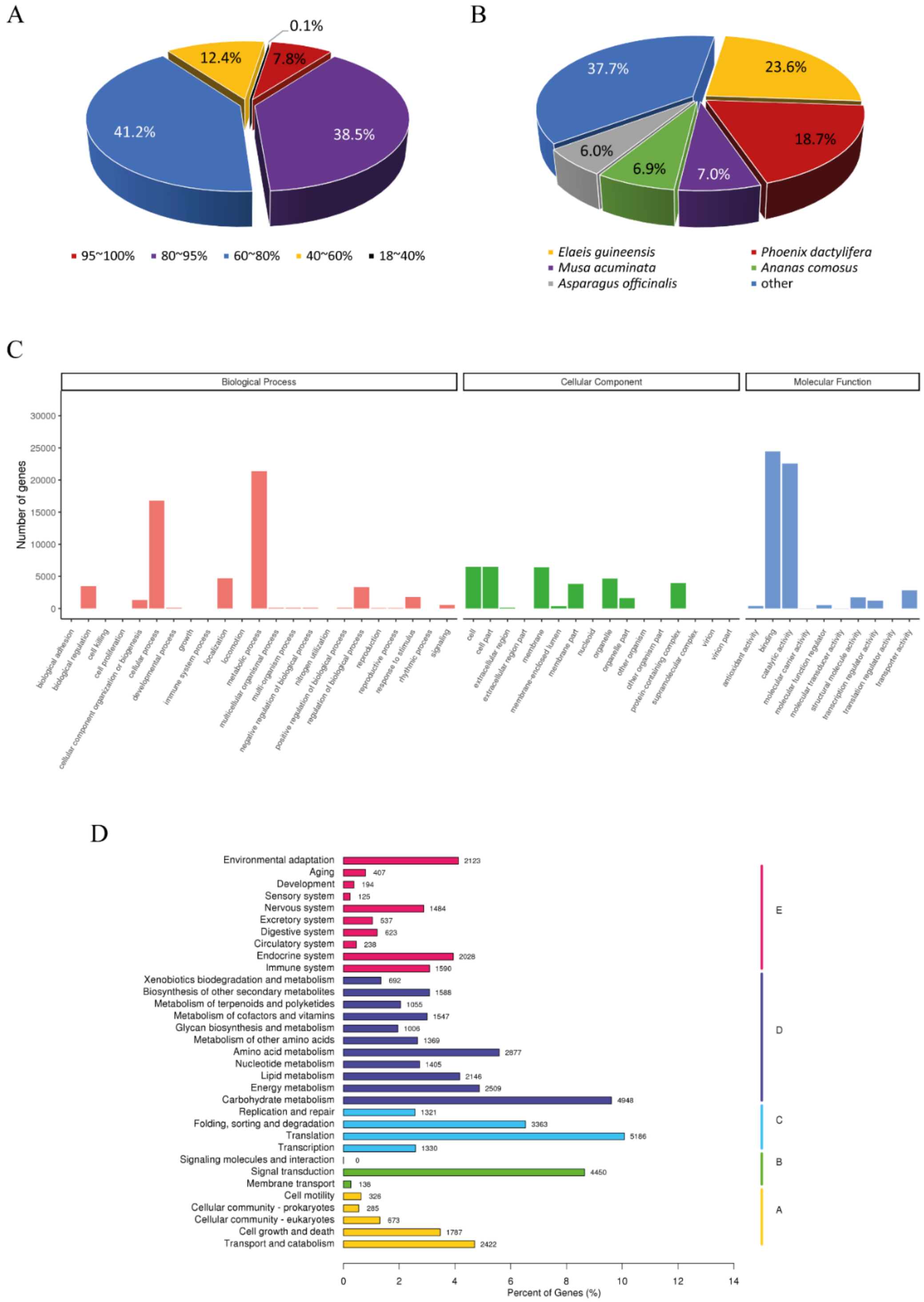

Supplementary Figure 2. Distribution and classification of unigenes. (A) BLAST similarity distribution. (B) BLAST top-hit species distribution based on NR annotation. (C) GO term classification for the domains biological process, cellular component and molecular function. (D) KEGG classification showing specific KEGG pathways.

1.2 Supplementary Tables

Supplementary Table 1 Primer used for gene expression analysis by quantitative real-time PCR.

Gene	Gene ID	Sequence (5′→3′)
DpEIN2	Cluster-14369.70879	GCAACTGGATTAGCAATATGC
		CAGCCAAGTATAGGGTGAGG
DpGa2ox4	Cluster-14369.105151	ACCACTCCTACCCACCC
		GAGGGCATCGTCGTCTAG
DpIDD7	Cluster-14369.110834	TTGTAATGTGCTTGAAGACTTAG
		TTATGCAACCATTGGTGAG
DpCYP90D2	Cluster-14369.98779	TCCCCTCTTCTCAAGTCC
		TCCAATCAAGACTCTCACC
DpFT8	Cluster-13468.4	CCCTGATGCACCAAGTCCAA
		TTCCTGTCACTGCTCTTGGG
DpSMT1	Cluster-14369.30153	CTTCCGTTTGACAGCATTC
		CCTTCCTTCCACCTTCC
DpBSK1-2	Cluster-14369.125260	AACGCCCTTCAAGATGCC
		GCTCTTCTAGTGTAGTTGCTTC
DpSKD1a	Cluster-14369.69556	CTTTTGTATGGTCCTCCTGGAAC
		ATTTTGAAACTAAGTCTGAGGAAG
DpSKD1b	Cluster-14369.87157	CTTTTGTATGGTCCTCCTGGAAC
		AGTTTGAGACTAAGTCCGAGGAAG
DpDWF1	Cluster-14369.88130	CGCCCCAAAAGAAAGAAG
		GTCGCCAAGATATAACAAGAAG
DpDWF4	Cluster-14369.135154	CCTCCATCCCTATAAACTTCC
		CCTTTCTTCACTTTCTCCAC
DpEXO	Cluster-14369.87832	GTCCACCGCCGAGCCTAGCG
		AGCGATTTCCCAAGCGAGCACT
DpOFP1	Cluster-14369.44426	AAGAAGAGACTTGGTAAAGCTC
		GTTGTGCACTAGTTCTTCTC
DpOFP6	Cluster-14369.127960	CCATTCCCATTTCCATTCCCATC
		TCGCTCACCTTCACCACC
DpF26G1	Cluster-14369.36214	GCTCGCAGATTCTTGGTTGTT
		TCCGACTTGTCCTTCTTGTGTTC
DpGIL1	Cluster-14369.130987	CGGAGTTCCGGCGAGATAG
		GACTCCTCCATCTTCGCGTG
DpTTL1	Cluster-14369.9088	CCCTCAAAATCCAACCAAAC
		CCTTCCTAAGCACTTCATAGTC
DpCUC3	Cluster-17788.0	CTGGTTTTTTATAGTGGAAGAGC

		TCACTCTCTCCTCTTTCCTC
DpIAA17	Cluster-14369.105333	GAATACCTGGCAGAGATGGAC
		CCTCCTACAGGAATCAGTGAAC
DpEXPA1	Cluster-20273.0	AGCCAAGGATATGGAACAAAC
		CACCATTTAGCATCATTGACAC
DpEXPA4	Cluster-14369.123030	TGTTCAACGATGGGTTCAG
		GAAGTTAGGAGGGCAGAAG
DpTCH4	Cluster-14369.136735	TCTTCATACCAATGTGTTCG
		TTTGGGGTTCCAAACAATG
DpIAA17	Cluster-14369.105333	GAATACCTGGCAGAGATGGAC
		CCTCCTACAGGAATCAGTGAAC
DpSAUR50	Cluster-18276.0	TCACAAGGGATGGATGATGAG
		GAAGAGACTGGAACTGAGGATG
DpTMK4	Cluster-39194.0	ACAATGCGCCCTATGAC
		CCACACCAAATGCATACAC
DpTIP41	Cluster-14369.94060	TCTCTTGCATGTTGAAAGTGGC
		GCTGAGAAGGTTTGCTCCTG
DpTUB	Cluster-14369.71430	CTTTGTGTTTGGGCAGTCT
		CACAGTTCTCAGCCTCCTT

Supplementary Table 2 Summary of QC analysis of biological replicates.

Sample	Clean reads	Clean data (Gb)	Q20 (%)	Q30 (%)	GC content(%)
F60H_1	60,640,954	18.2	98.36	94.92	46.4
F60H_2	59,097,779	17.7	98.38	94.87	46.62
F60H_3	57,467,552	17.2	98.36	94.92	46.71
F60M_1	56,815,642	17	98.39	94.93	45.32
F60M_2	64,120,441	19.2	98.48	95.1	45.34
F60M_3	65,941,873	19.8	98.5	95.12	45.36
F60T_1	55,611,580	16.7	98.43	95	46.02
F60T_2	72,044,504	21.6	98.23	94.47	46.53
F60T_3	79,141,263	23.7	98.39	94.98	46.58
F2000H_1	61,253,593	18.4	98.03	94.39	47.04
F2000H_2	64,790,042	19.4	98.07	94.45	47.18
F2000H_3	70,133,425	21	98.08	94.43	47.58
F2000M_1	66,819,024	20	98.02	94.27	45.79
F2000M_2	57,458,193	17.2	98.06	94.4	45.81
F2000M_3	74,185,907	22.3	98.41	94.94	45.69
F2000T_1	59,049,216	17.7	98.37	94.93	46.68
F2000T_2	58,505,147	17.6	98.37	94.85	46.42
F2000T_3	55,464,040	16.6	98.3	94.66	46.42
