# Supplementary material for: Transcriptomic analysis of Chinese yam (Dioscorea polystachya Turcz.) variants indicates brassinosteroid involvement in tuber development
Source: Front Nutr. 2023 May 5;10:1112793. doi: 10.3389/fnut.2023.1112793 (PMC10196131; doi:10.3389/fnut.2023.1112793)
Supplement: Supplementary file 3 [file Data_Sheet_3.pdf]

Supplementary Methods

Transcriptomic analysis of Chinese yam (*Dioscorea polystachya* Turcz.) variants indicates brassinosteroid involvement in tuber development

Jenny Riekötter, Jana Oklestkova, Jost Muth, Richard M. Twyman and Janina Epping *

*** Correspondence:** Janina Epping: Janina.epping@uni-muenster.de

1 Supplementary methods

1.1 RNA extraction from tuber material

Total RNA was extracted from tuber samples as previously described with modifications (1). We mixed 20 mg freeze-dried tuber powder with 400 µL TLES extraction buffer (100 mM Tris-HCl, pH 8.0, 10 mM EDTA, 0.1% (w/v) SDS, 0.1 M LiCl, 2% (w/v) PVPP, 40 mM DTT) in a 2-mL reaction tube and vortexed for 1 min. Samples were chilled on ice and an equal amount of buffer-saturated phenol:chloroform:isoamyl alcohol (25:24:1, pH 5) was added followed by vigorous mixing. Tubes were centrifuged (15,800 *g*, 10 min, 4 °C) and the clear aqueous supernatant was transferred to a new sterile 1.5-mL reaction tube. An equal volume of 100% molecular biology-grade ethanol was carefully mixed with the supernatant. The mixtures were transferred to the second (pink) RNeasy mini spin column from the RNeasy Plant Mini Kit (Qiagen, Hilden, Germany) and the manufacturer's protocol was followed from the DNase digestion step. RNA quality was checked by 1% (w/v) agarose gel electrophoresis, and RNA purity and integrity were tested at OD_{260}/OD_{280} using a BioSpectrometer basic (Eppendorf, Hamburg, Germany) and Agilent 2100 Bioanalyzer System (Agilent Technologies, Santa Clara, CA, USA), respectively.

1.2 Library construction and Illumina sequencing

Strand-specific mRNA libraries were constructed and sequenced by Novogene (Beijing, China). Libraries were prepared from three biological replicates per F60 and F2000tuber part using the NEBNext Ultra RNA Library Prep Kit for Illumina. The mRNA was enriched from 1 µg of total RNA using oligo(dT) magnetic beads. After random fragmentation and cDNA synthesis, the second strand was generated by nick translation using Illumina custom second-strand synthesis buffer supplemented with dNTPs, RNase H and *Escherichia coli* polymerase I. Sequencing adapters were ligated to the cDNA and, after size selection and PCR enrichment, the concentration of the final cDNA libraries was determined using a Qubit 2.0 fluorometer (Thermo Fisher Scientific, Waltham, MA, USA). Insert size was confirmed on an Agilent 2100 Bioanalyzer (dilution to 1 ng/µL) and by quantitative PCR (qPCR). Final cDNA libraries were sequenced on a NovaSeq 6000 platform with an S4 flow cell (Illumina, San Diego, CA, USA).

1.3 DNA extraction and analysis of the genetic relationship between *D. polystachya* cultivars

Genomic DNA was extracted as previously described with modifications (2). Briefly, 1 mL ice-cold CTAB-free buffer (200 mM Tris-HCl pH 8.0, 50 mM EDTA, 250 mM NaCl) and 6 µL precooled 2-mercatpoethanol were mixed with 30 mg tuber powder pooled from three individuals from each cultivar in a 2.0-mL tube and incubated on ice for 10 min. After centrifugation (10,000 *g*, 10 min, 4 °C), the supernatant was discarded (if viscous, the previous step was repeated) and the pellet was redissolved in 500 µL prewarmed (65 °C) CTAB buffer (100 mM Tris-HCl pH 8.0, 25 mM EDTA, 1.5 M NaCl, 3% (w/v) CTAB, 1% (w/v) PVP, 1% (w/v) SDS) containing 5 µL 2-mercaptoethanol. After incubation at 65 °C for 60 min, the tubes were cooled to room temperature and we added 0.5 volumes of chloroform:isoamyl alcohol (24:1 (v/v)). After mixing by inversion for 1 min, samples were centrifuged (16,000 *g*, 15 min, room temperature) and the upper aqueous phase was collected in a new 1.5-mL tube. The previous step was repeated until the aqueous layer became clear. Then, we added 0.5 volumes of 5 M NaCl and 1 volume of ice-cold isopropanol and incubated at –20 °C overnight. DNA was pelleted by centrifugation (16,000 *g*, 15 min, 4 °C) and the supernatant was discarded. The pellet was washed twice with 1 mL 75% (v/v) ethanol each followed by a centrifugation step (16,000 *g*, 5 min, 4 °C). After drying the pellet at room temperature, we added 400 µL high-salt TE (1 M NaCl in TE) and 2 µL RNase A (Thermo Fisher Scientific) and incubated at 37 °C for 60 min with occasional mixing to ensure the pellet was completely dissolved. DNA was precipitated by adding 2 volumes of ice-cold 100% ethanol and incubating at –20 °C for 60 min. The sample was centrifuged (16,000 *g*, 15 min, 4 °C) and the pellet was washed twice with 75% (v/v) ethanol. The air-dried pellet was dissolved in 100 µL TE. Genomic DNA was extracted from tuber shape variants F60, F2000, Genkotsu-Jiro, Zenguritaro, Shintammaru, Tamba-Yamatoimo, DpYam21 and *D. rotundata* cv. TDr 95/19177. Tubers of Genkotsu-Jiro, Zenguritaro, Shintammaru and Tamba-Yamatoimo were imported from a yam provider in Japan. DpYam21 was provided by the Genebank department of the IPK Leibnitz Institute, Gatersleben, Germany. *D. rotundata* TDr 95/19177 was kindly provided by the National Root Crops Research Institute, Umudike, Nigeria. To determine the genetic relationship between the variants, ISAP markers were used as previously described (3) with slight modifications. Briefly, each reaction comprised 1 µL DNA (25 ng/µL), 4 µL 5× Mango*Taq* Colored Reaction Buffer (Bioline, Luckenwalde, Germany), 0.5 U Mango*Taq*, 2.5 mM $MgCl_2$, 0.5 pmol of each primer, 0.5 mM dNTPs and 10.9 µL ddH_2O. The following program was run on a Mastercycler nexus (Eppendorf, Hamburg, Germany): 95 °C for 5 min, followed by five cycles of 95 °C for 1 min, 36 °C for 1 min and 72 °C for 1 min, then 35 cycles of 95 °C for 1 min, specific annealing temperature of each primer pair for 1 min and 72 °C for 1 min, and a final elongation step of 72 °C for 10 min.

1. Bömer M, Rathnayake AI, Visendi P, Sewe SO, Sicat JPA, Silva G, Kumar PL, Seal SE. Tissue culture and next-generation sequencing: A combined approach for detecting yam (*Dioscorea* spp.) viruses. *Physiol Mol Plant Pathol* (2018)1–13. doi: 10.1016/j.pmpp.2018.06.003

2. Zeng J, Zou YP, Bai JY, Zheng HS. Preparation of Total DNA from “Recalcitrant Plant Taxa.” *Acta Bot Sin* (2002) 44:694–697.

3. Peng B, Zhang Y, Sun X, Li M, Xue J, Hang Y. Genetic relationship and identification of *Dioscorea polystachya* cultivars accessed by ISAP and SCAR markers. *Arch Biol Sci* (2017) 69:277–284. doi: 10.2298/ABS150717098P
